# Supplementary material for: Efficient hydrogen production on MoNi4 electrocatalysts with fast water dissociation kinetics
Source: Nat Commun. 2017 May 17;8:15437. doi: 10.1038/ncomms15437 (PMC5442356; doi:10.1038/ncomms15437)
Supplement: Supplementary Information — Supplementary Figures, Supplementary Table, Supplementary Note and Supplementary References [file ncomms15437-s1.pdf]

Supplementary Note 1.

Electrocatalytic HER mechanisms in alkaline and acidic solutions:

HER in alkaline solution:

$H_2O + e^- + cat \rightarrow H^*\text{-}cat + {}^-OH$ Volmer step

$2H^*\text{-}cat \rightarrow \uparrow H_2$ Tafel step

$H^*\text{-}cat + H_2O + e^- \rightarrow cat + {}^-OH + \uparrow H_2$ Heyrovsky step

HER in acidic solution:

$H^+ + e^- + cat \rightarrow H^*\text{-}cat$ Volmer step

$2H^*\text{-}cat \rightarrow \uparrow H_2$ Tafel step

$H^*\text{-}cat + H^+ + e^- \rightarrow cat + \uparrow H_2$ Heyrovsky step

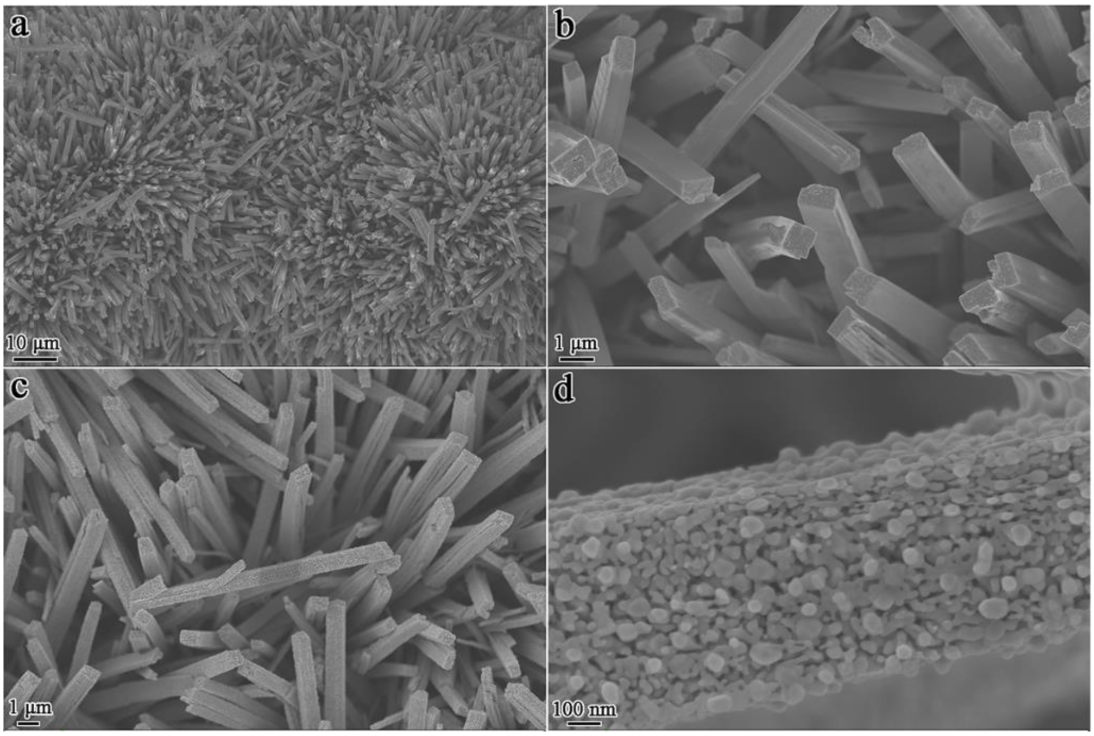

Supplementary Figure 1 | Morphology. SEM images of the $NiMoO_4$ and $MoNi_3$ nanoparticles on the MoO_2 after $NiMoO_4$ cuboids on the nickel foam are calcined for 2 h at different temperatures in a H_2/Ar atmosphere: (a and b) $NiMoO_4$ cuboids at 400 °C and (c and d) $MoNi_3$ nanoparticles at 600 °C.

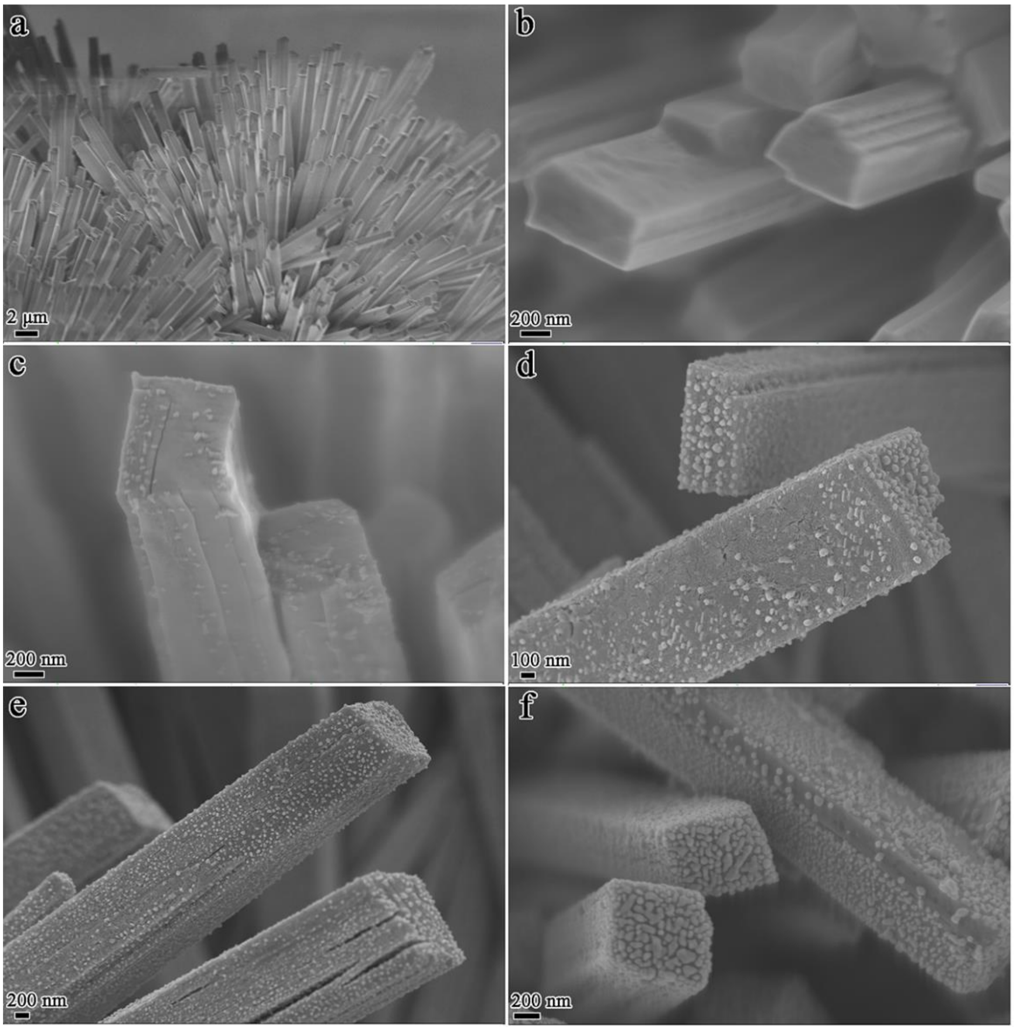

Supplementary Figure 2 | Morphology. SEM images of the $MoNi_4$ cuboids when $NiMoO_4$ cuboids on the nickel foam are calcined for different lengths of time at 500 °C in a H_2/Ar atmosphere: (a) 0 min; (b) 15 min; (c) 30 min; (d) 1 h; (e) 2 h and f) 4 h.

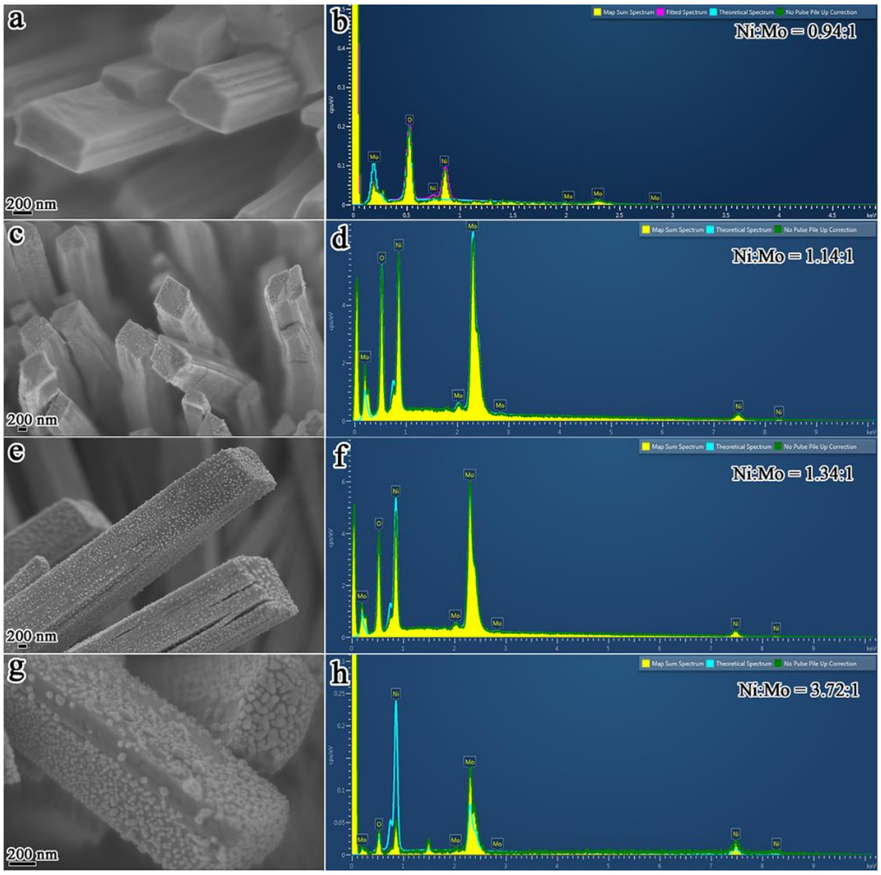

Supplementary Figure 3 | Morphology and chemical composition analyses. SEM images and corresponding EDX analyses of the $MoNi_4$ cuboids when $NiMoO_4$ cuboids on the nickel foam are calcined for different lengths of time at 500 °C in a H_2/Ar atmosphere: a-b) 0 h; c-d)1 h; e-f) 2 h; g-h) 4 h. Along with the increase of calcination time from 0 h to 4 h, the molar ratio of Ni to Mo is augmented from initial 0.94:1 to 3.72:1 as a result of the aggregation of $MoNi_4$ on the surfaces of MoO_2 cuboids.

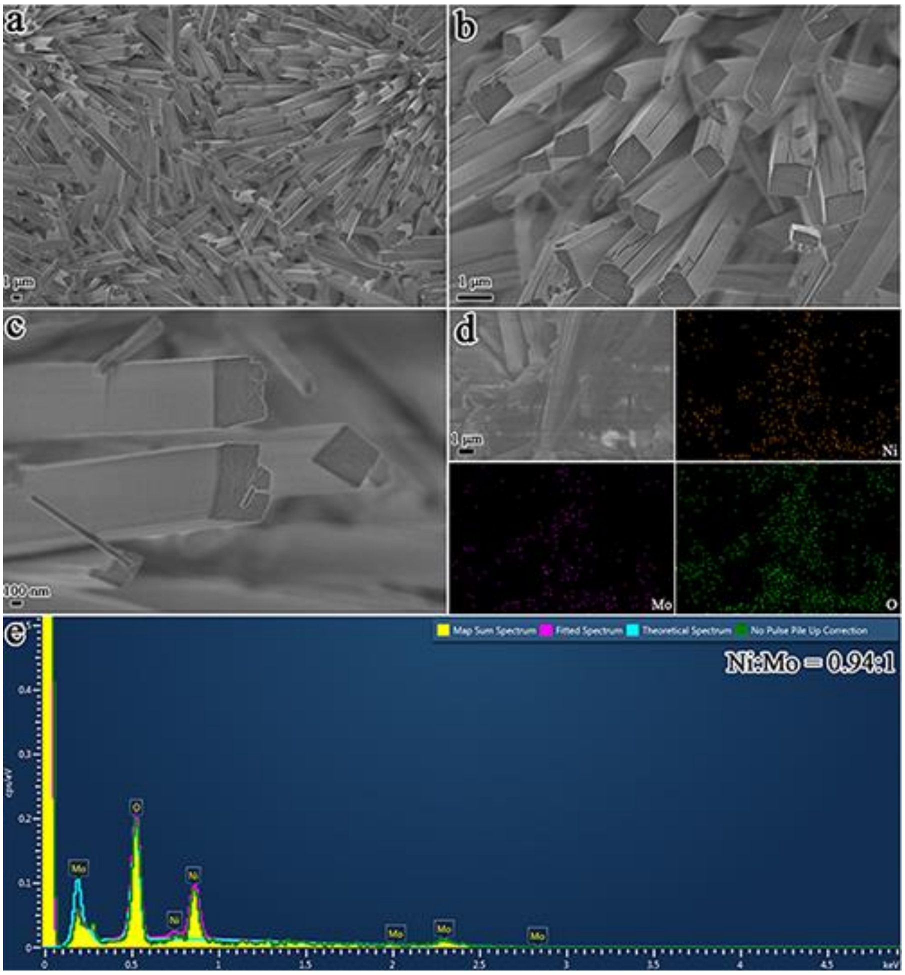

Supplementary Figure 4 | Morphology and chemical composition analyses. (a-c) SEM images of the $NiMoO_4$-Ar when the $NiMoO_4$ cuboids on the nickel foam were heated for 2 h in Ar atmosphere; d) Corresponding elemental mapping images of Ni, Mo and O and e) related EDX analysis. Only vertically-aligned cuboids with smooth surfaces are observed on the nickel foam. The cuboids are composed of Ni, Mo and O elements and the molar ratio of Ni to Mo is approximately 0.94:1.

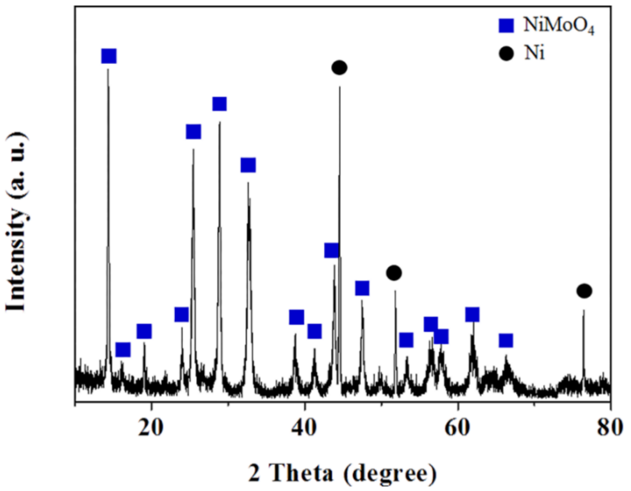

Supplementary Figure 5 | The XRD pattern of the $NiMoO_4$-Ar when the $NiMoO_4$ cuboids on the nickel foam were heated for 2 h at 500 °C in Ar atmosphere. The XRD analysis confirms that the obtained cuboids are $NiMoO_4$. This result proves that that H_2 gas is a key parameter for the construction of $MoNi_4/MoO_2$@Ni.

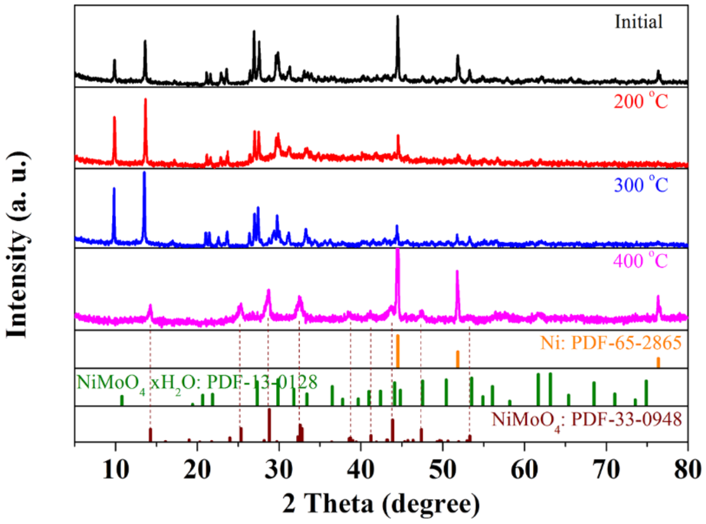

Supplementary Figure 6 | The XRD patterns of the $NiMoO_4$ on the nickel foam after treatment at different temperatures in Ar atmosphere. The as-obtained $NiMoO_4$ cuboids on the nickel foam were dried for 12 h at 80 °C in an electric oven. Thus, the crystalline hydrated H_2O molecules still exist as $NiMoO_4$•xH_2O. The achieved sample thus consists of $NiMoO_4$ and $NiMoO_4$•xH_2O. In order to remove the crystalline water from $NiMoO_4$•xH_2O, the initial $NiMoO_4$@Ni was heated at increased temperature from 200 to 400 °C in Ar atmosphere. Clearly, the XRD diffraction peaks of the obtained sample at 400 °C accord perfectly with the standard peaks of the $NiMoO_4$ (PDF-33-0948). This result suggests that the crystalline water molecules have been removed.

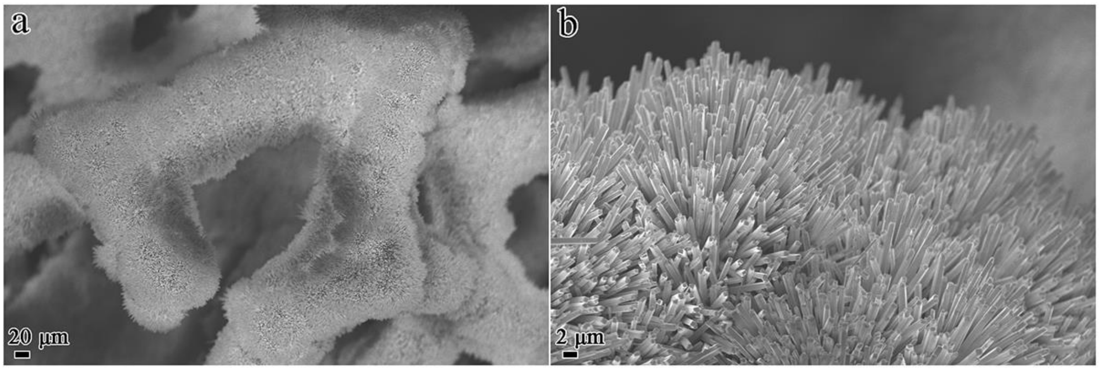

Supplementary Figure 7 | Morphology. SEM images of the as-constructed $NiMoO_4$ cuboids on the nickel foam. The loading weight of the $NiMoO_4$ cuboids on the nickel foam is estimated to be 55.6 mg cm^2.

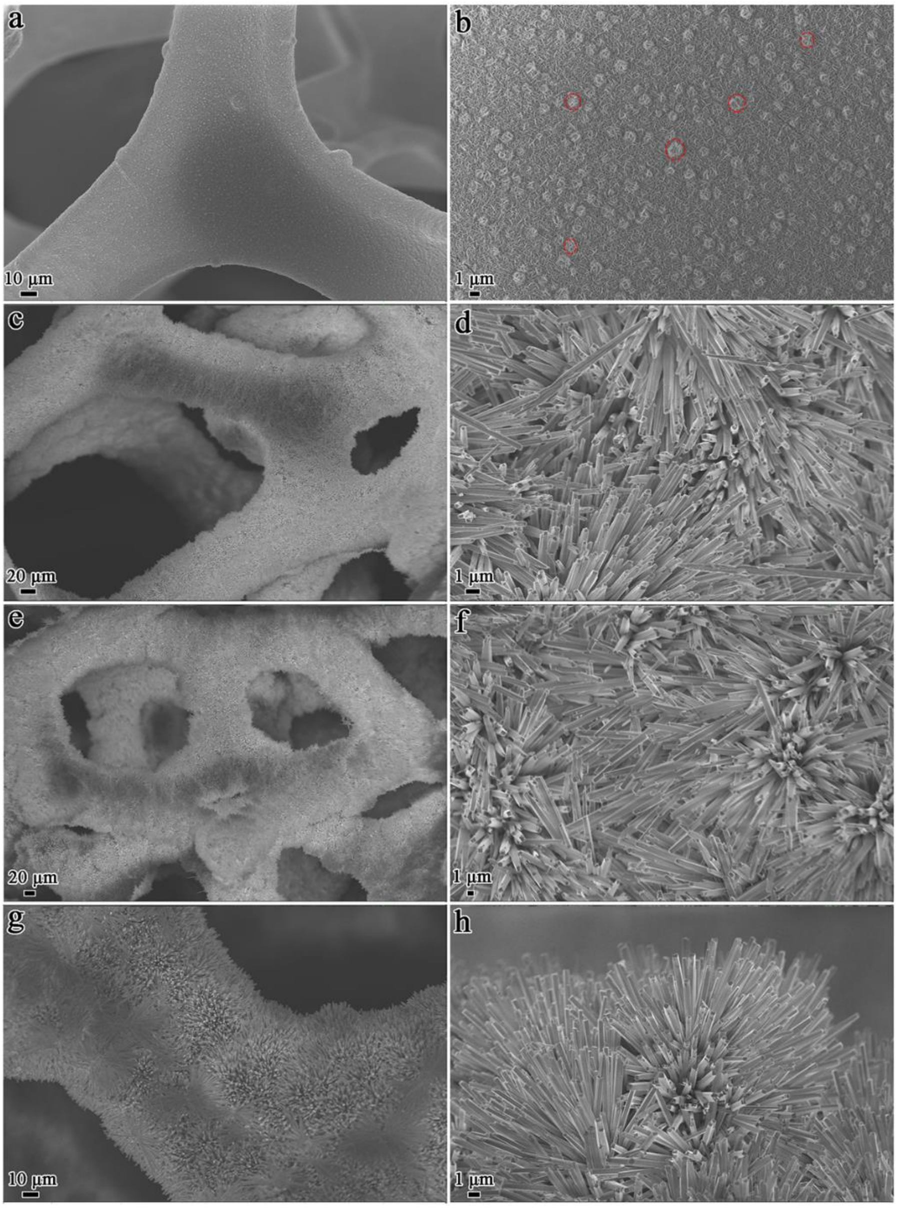

Supplementary Figure 8 | Morphology. SEM images of the as-obtained $NiMoO_4$ cuboids on the nickel foam at different hydrothermal reaction times: (a and b) 0.5 h, (c and d) 1 h, (e and f) 2 h and (g and h) 4 h.

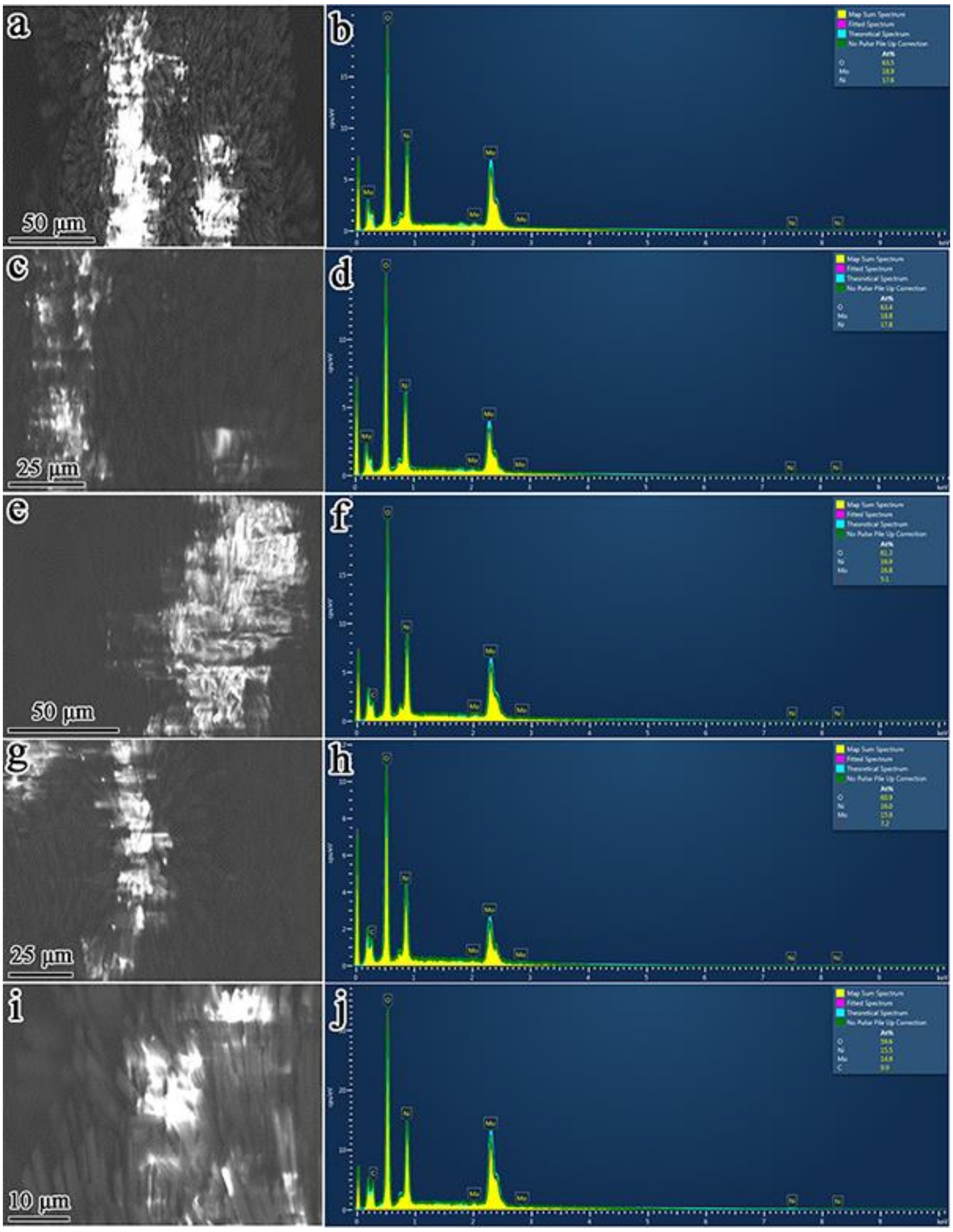

Supplementary Figure 9 | Chemical composition analyses. (a) SEM images and corresponding EDX spectra of the $NiMoO_4$@Ni. the average atomic ratio of Ni to Mo is about 1: 1.01.

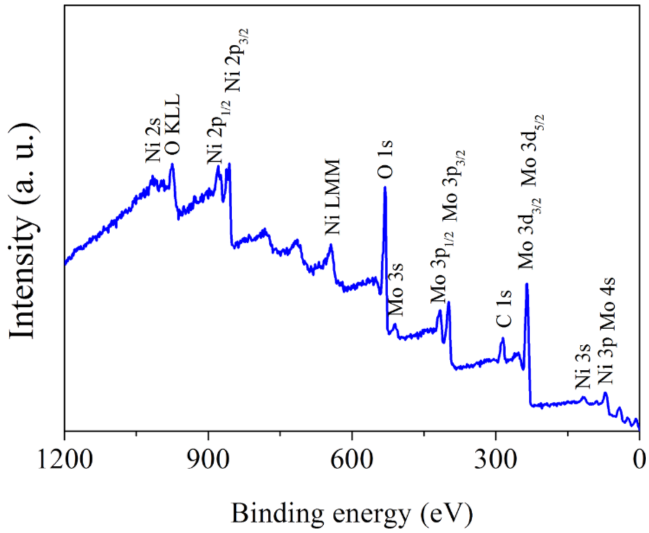

Supplementary Figure 10 | The XPS pattern of the as-obtained $NiMoO_4$ cuboids on the nickel foam.

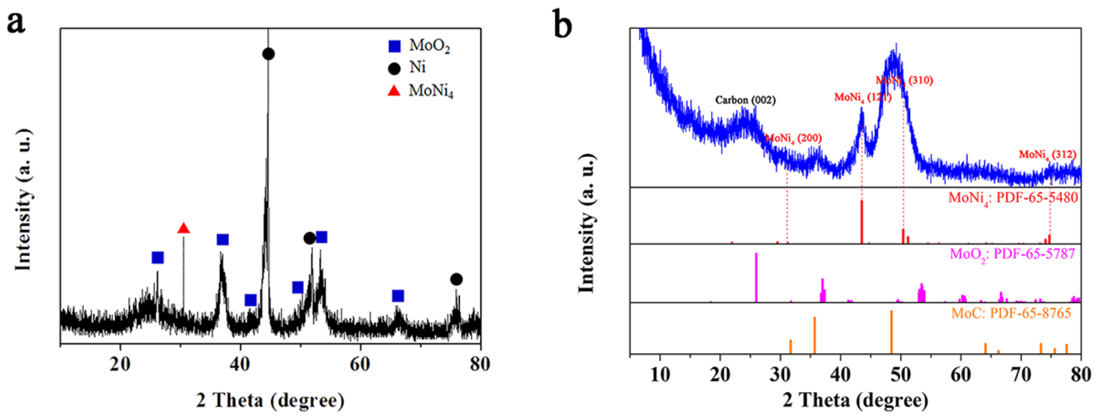

Supplementary Figure 11 | The XRD patterns of a) $MoNi_4/MoO_2$@Ni and b) $MoNi_4/MoO_2$@C.

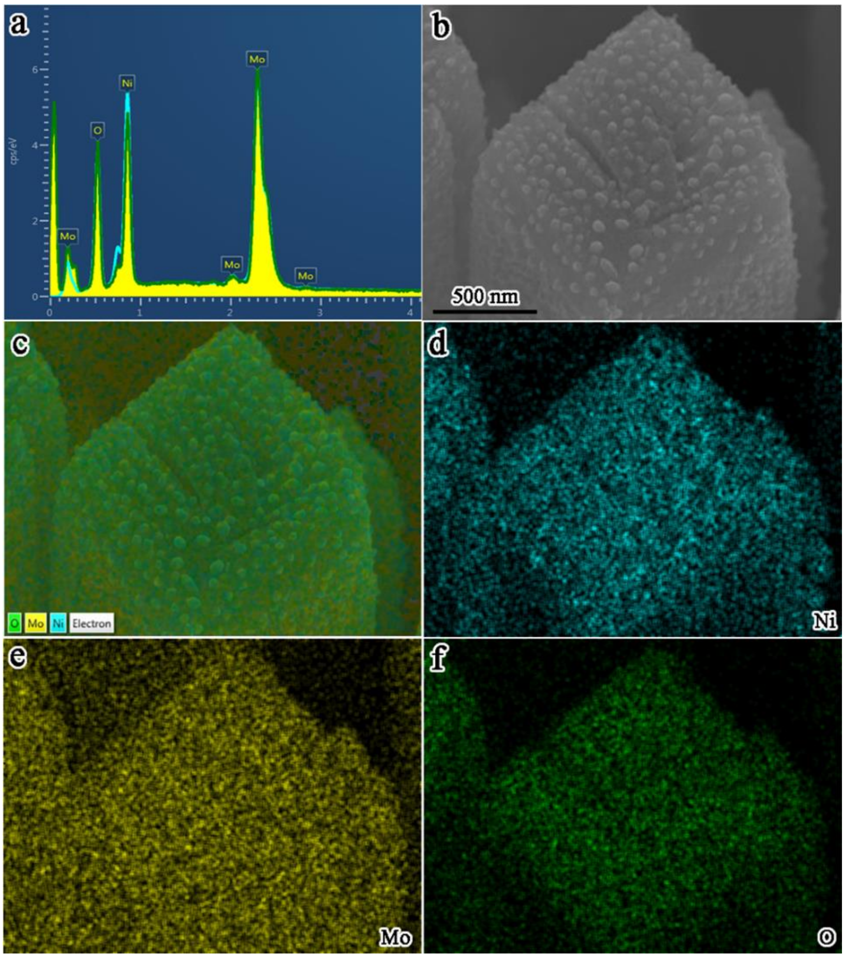

Supplementary Figure 12 | Morphology and chemical composition analyses. (a) EDX analysis, (b) SEM image and (c-f) corresponding elemental mapping images of as-constructed $MoNi_4/MoO_2@Ni$.

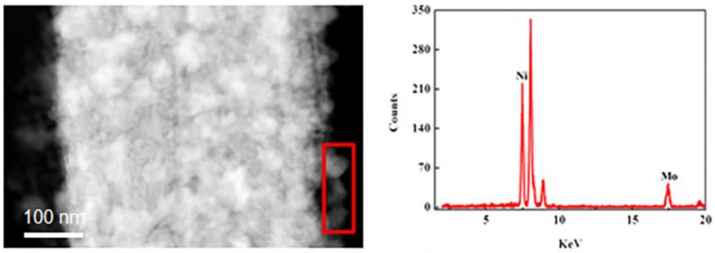

EDS Analysis of Extracted Spectrum 2

Quantification Mode: Standard Less

Correction Mode: Cliff Lorimer

Fit index (chi**2): 30.1

Number of iterations: 1

Total atoms: 5.0

Total weight: 100.0

Element	Line	KRatio	Correction	Atoms %
Ni	Ka	0.701?.0288	1.0000	79.32?.26
Mo	Ka	0.299?.0721	1.0000	20.68?.99

EDS Analysis complete.

Supplementary Figure 13 | The original EDX spectrum of the surface $MoNi_4$ nanoparticles.

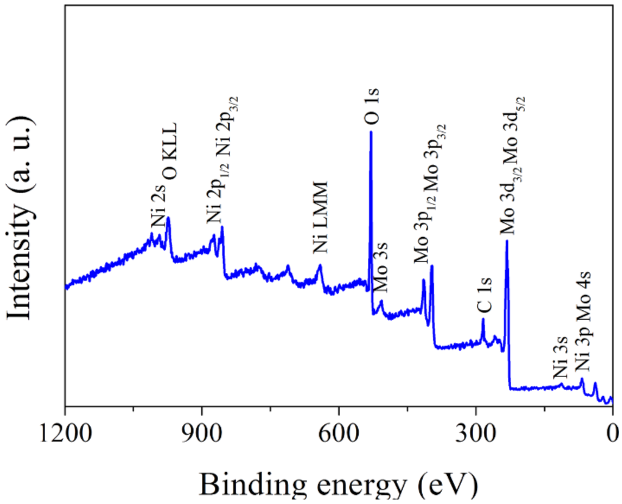

Supplementary Figure 14 | The XPS pattern of as-obtained $MoNi_4/MoO_2$@Ni. The $MoNi_4/MoO_2$@Ni is composed of Ni, Mo and O elements.

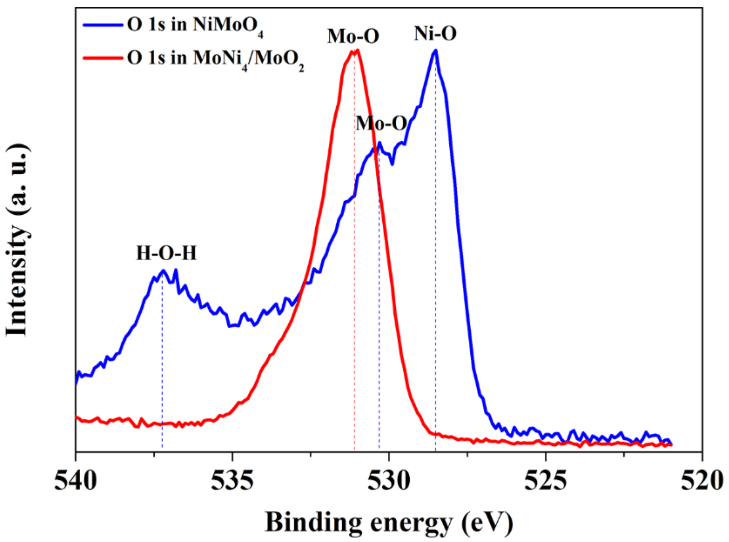

Supplementary Figure 15 | The high-resolution XPS pattern of O 1s for the $NiMoO_4$ cuboids and $MoNi_4/MoO_2$@Ni on the nickel foam. The O 1s peaks at 528.5 eV, 530.3 eV and 537.2 eV are assigned to Ni-O, Mo-O and H-O-H in $NiMoO_4$ cuboids, respectively. The O 1s signal of Mo-O in $MoNi_4/MoO_2$ appears at 531.1 eV.

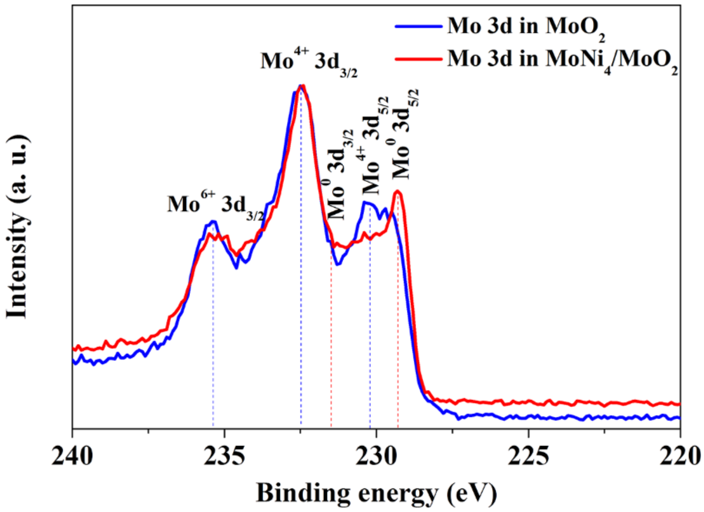

Supplementary Figure 16 | The high-resolution XPS pattern of Mo 3d for the MoO_2 cuboids and $MoNi_4/MoO_2$@Ni on the nickel foam. The peaks located at 230.2 eV, 232.5 eV, and 235.4 eV belong to Mo^{4+} $3d_{5/2}$, Mo^{4+} $3d_{3/2}$ and Mo^{6+} $3d_{3/2}$, respectively. The Mo^{0} $3d_{5/2}$ and Mo^{0} $3d_{3/2}$ are detected at 229.3 eV and 231.5 eV, respectively.

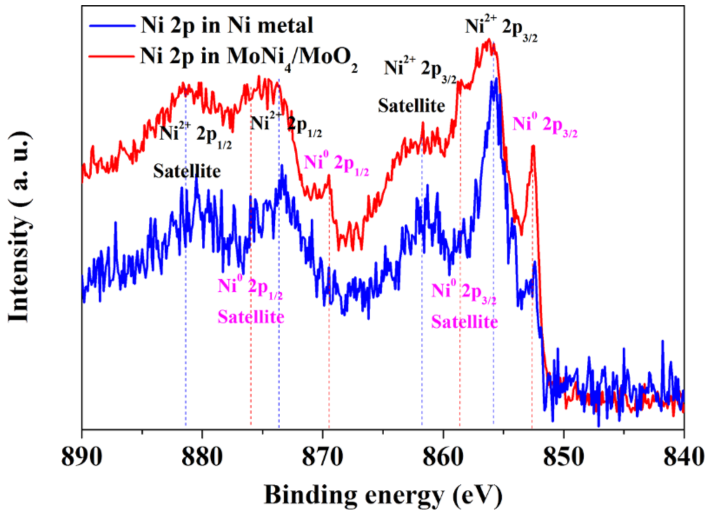

Supplementary Figure 17 | The high-resolution XPS pattern of Ni 2p for the Ni nanosheets and $MoNi_4/MoO_2$@Ni on the nickel foam. the peaks at 855.8 eV, 861.7 eV, 873.7 eV and 881.4 eV are ascribed to Ni^{2+} $2p_{3/2}$, Ni^{2+} $2p_{3/2}$ satellite, Ni^{2+} $2p_{1/2}$ and Ni^{2+} $2p_{1/2}$ satellite, respectively. Ni^{0} $2p_{3/2}$, Ni^{0} $2p_{3/2}$ satellite, Ni^{0} $2p_{1/2}$ and Ni^{0} $2p_{1/2}$ satellite are observed at 852.6 eV, 858.5 eV, 869.5 eV and 876.0 eV, respectively.

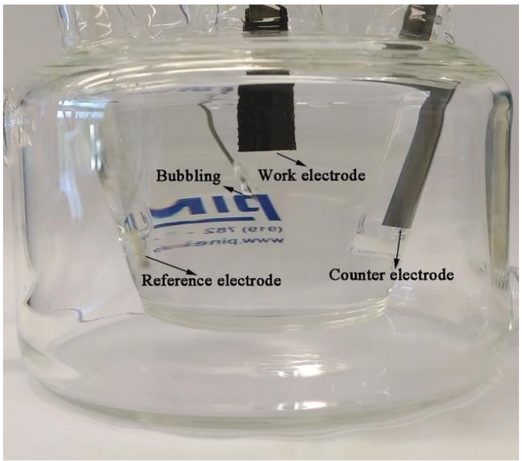

Supplementary Figure 18 | A three-electrode system for electrocatalytic HER tests. The distance between the working electrode and the reference electrode is approximately 4 cm.

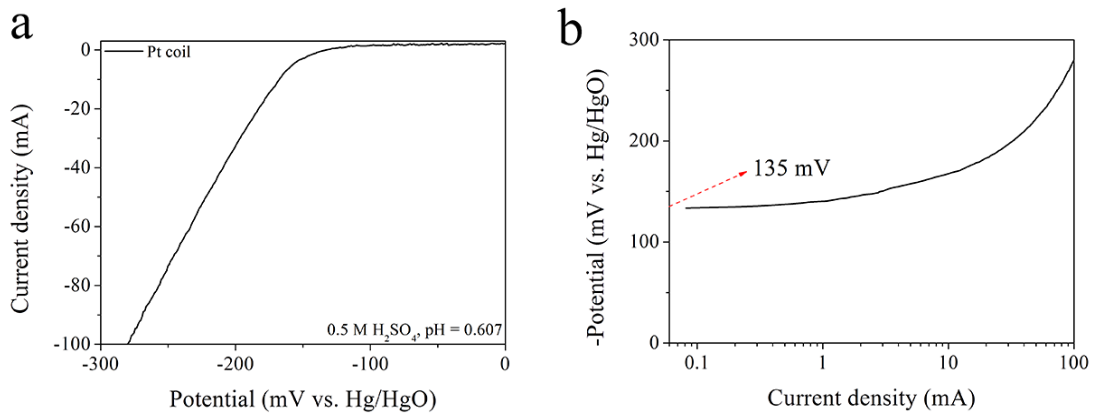

Supplementary Figure 19 | (a) Polarization curve and (b) corresponding Tafel plot of a Pt coil in a H_2SO_4 aqueous solution (pH = 0.607). The standard potential (vs. RHE) of the Hg/HgO reference electrode is calibrated to be approximately 0.099 V.

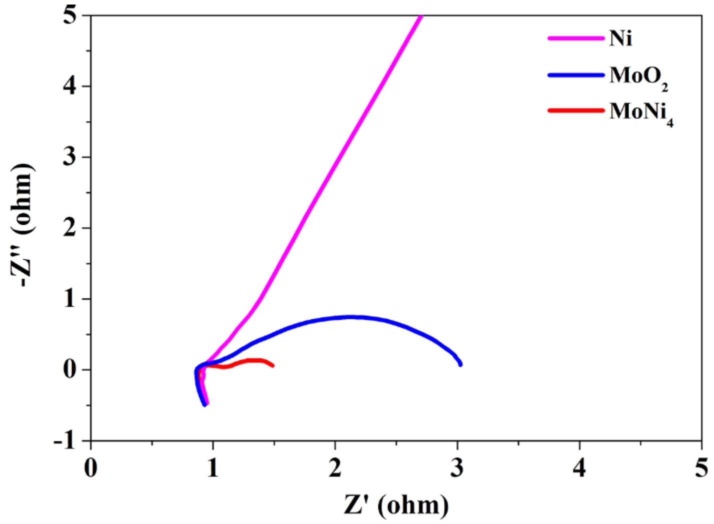

Supplementary Figure 20 | Electrochemical impedance spectroscopy (EIS) analyses of the catalysts. the electrochemical impedance spectroscopy (EIS) of catalysts have been measured in Ar-saturated 1 M KOH aqueous solution at -0.1 V vs. RHE with 10 mV AC potential from 10 kHz to 0.01 Hz.

The recorded impedances were presented in the form of imaginary (Im) vs. real (Re) parts at various frequencies. It has been recognized that the high frequency interception of the Re-axis represents the resistance of the electrodes. The width of the semicircle on the Re-axis corresponds to the charge-transfer resistances and indicates the overall kinetic effects. As indicated in Supplementary Figure 20, all catalysts exhibited almost the similar intrinsic resistance (~0.94 ohm), while the charge-transfer resistance of the $MoNi_4/MoO_2$@Ni electrocatalyst was much lower than those of the MoO_2 and Ni electrocatalysts, suggesting a faster HER kinetic process on the $MoNi_4/MoO_2$@Ni elelctrocatalyst.

The ohmic potential drop (iR_s) loss from the electrolyte resistance was subtracted according to the following equation:

$P_{vs.\ RHE} = P_{vs.\ Hg/HgO} + 0.059*pH - i * R_s$

$P_{vs.\ RHE}$ is the potential versus standard hydrogen electrode. $P_{vs.Hg/HgO}$ is the measured potential with Hg/HgO electrode as reference electrode. R_s is the electrolyte resistance. The pH value of 1 M KOH aqueous solution is about 13.97. i is the recorded current density.

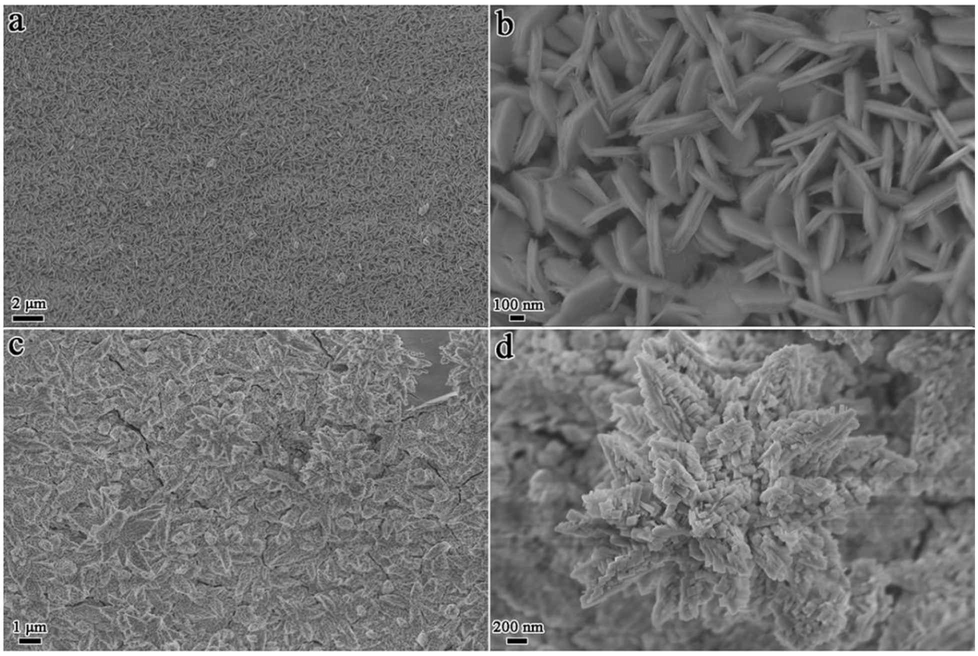

Supplementary Figure 21 | Morphology. SEM images of (a and b) $Ni(OH)_2$ nanosheets and (c and d) MoO_3 nanostructures on the nickel foam.

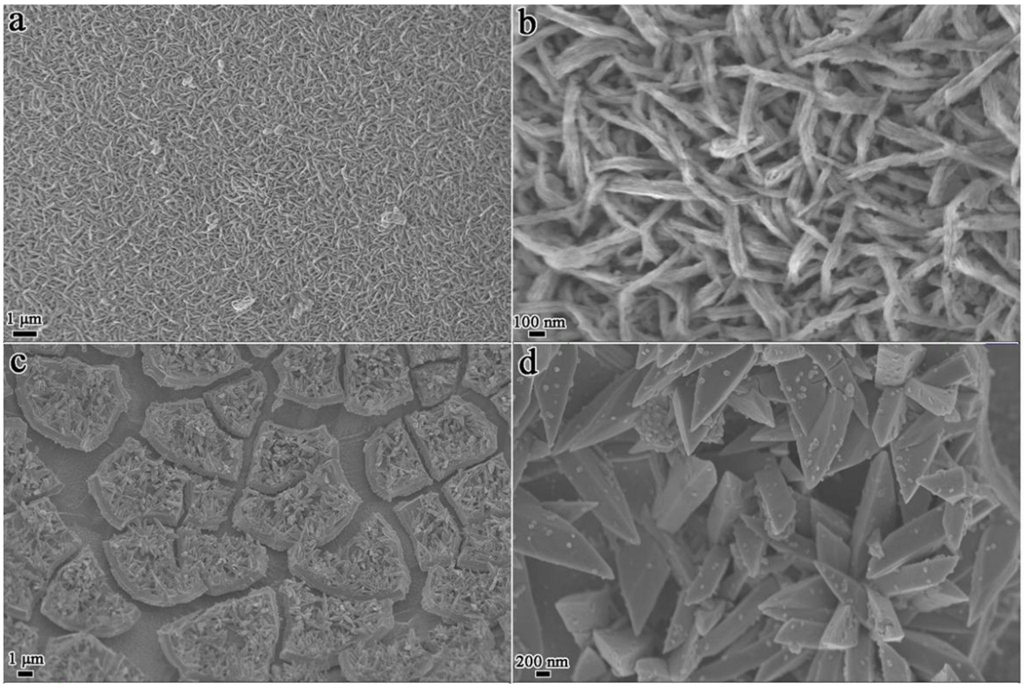

Supplementary Figure 22 | Morphology. SEM images of (a and b) Ni nanosheets and (c and d) MoO_2 cuboids on the nickel foam.

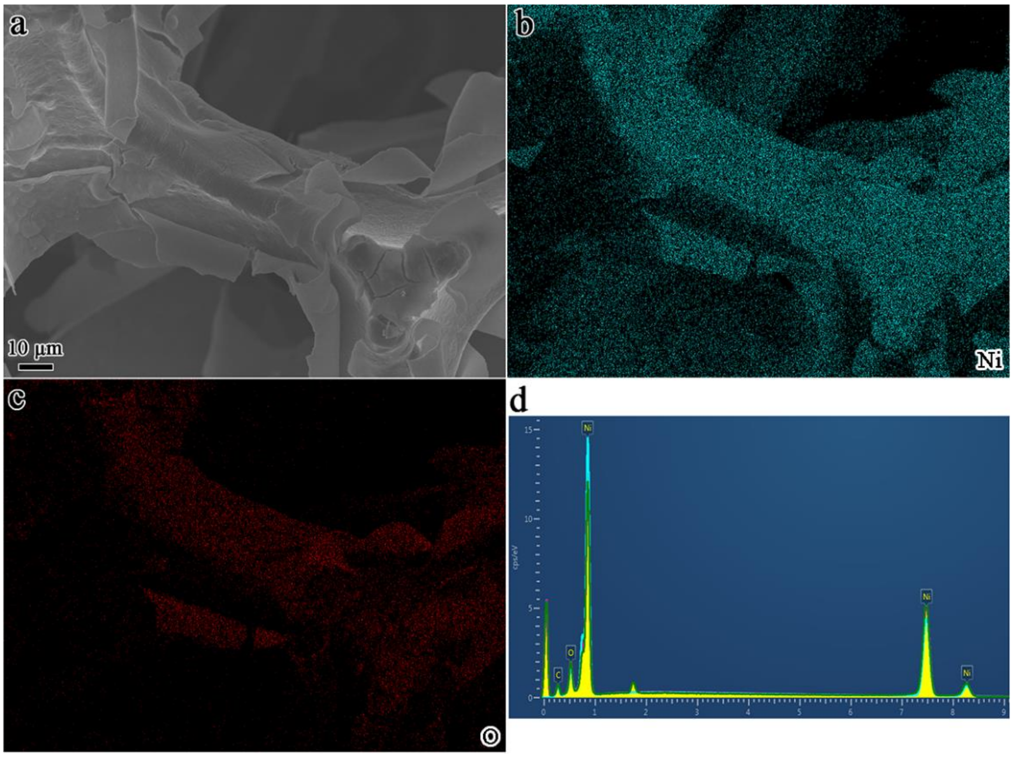

Supplementary Figure 23 | Chemical composition analyses. (a) SEM image of the as-constructed Ni nanosheets on the nickel foam; corresponding elemental mapping images: (b) Ni and (c) O; (d) EDX analysis of the as-constructed Ni nanosheets on the nickel foam.

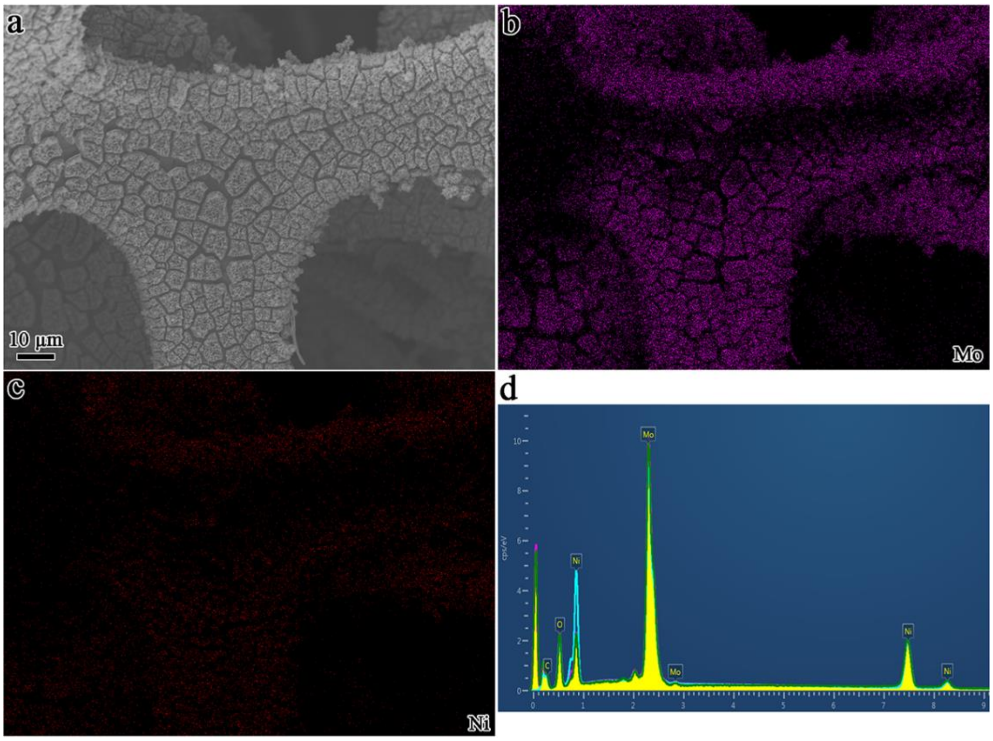

Supplementary Figure 24 | Chemical composition analyses. (a) SEM image of the as-constructed MoO_2 cuboids on the nickel foam; corresponding elemental mapping images: (b) Mo and (c) Ni; (d) EDX analysis of the as-constructed MoO_2 cuboids on the nickel foam.

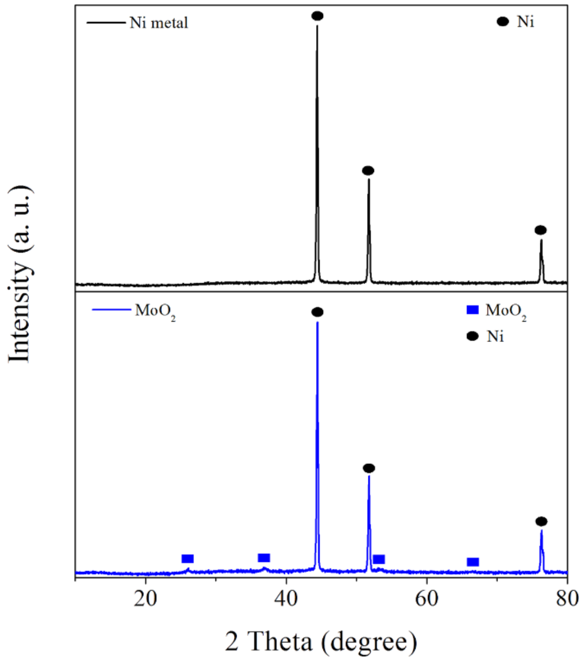

Supplementary Figure 25 | The XRD patterns of the as-obtained Ni nanosheets and MoO_2 cuboids on the nickel foam.

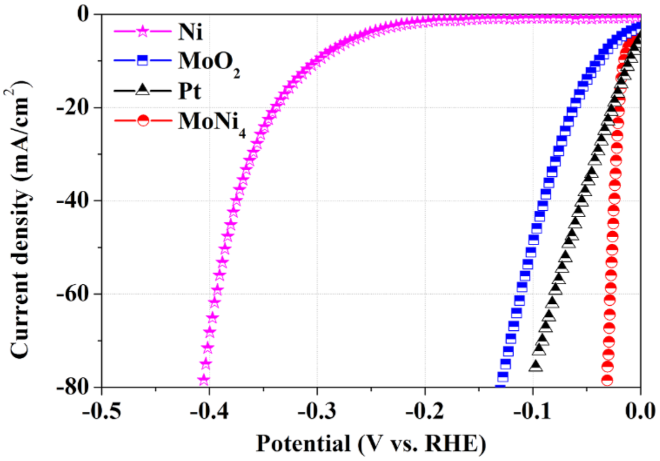

Supplementary Figure 26 | Polarization curves of the electrocatalysts in a 1 M KOH aqueous solution.

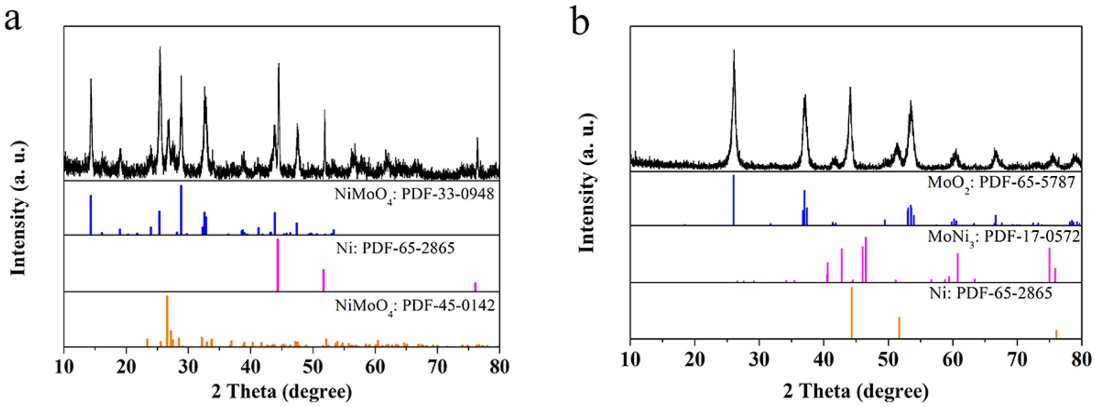

Supplementary Figure 27 | The XRD patterns of the samples after the calcination of $NiMoO_4$ cuboids at different temperatures in H_2/Ar atmosphere: a) 400 ^{o}C and b) 600 ^{o}C. The obtained sample at 400 ^{o}C is $NiMoO_4$ cuboids while the sample at 600 ^{o}C is $MoNi_3$ nanoparticles supported by the MoO_2.

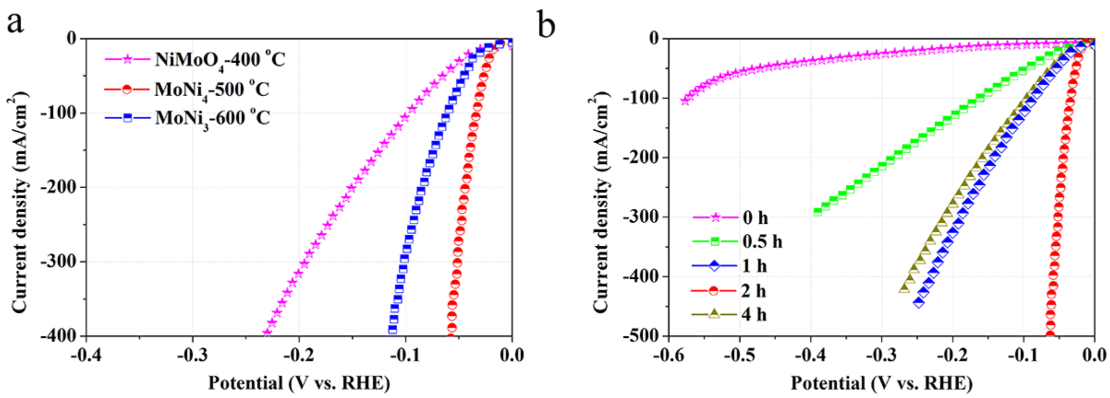

Supplementary Figure 28 | Electrochemical tests. a) Polarization curves of the $NiMoO_4$ cuboids, the $MoNi_3/MoO_2$@Ni cuboids and $MoNi_4/MoO_2$@Ni on the nickel foam; b) polarization curves of the products after the calcination of $NiMoO_4$ cuboids for different times at 500 °C in a H_2/Ar atmosphere. Electrolyte: 1 M KOH aqueous solution; LSV scan rate: 1 mV/s.

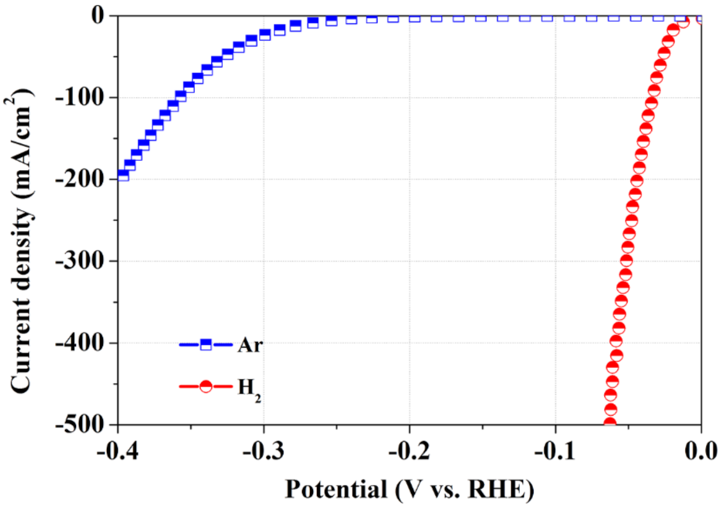

Supplementary Figure 29 | The polarization curves of the $NiMoO_4$-Ar when the $NiMoO_4$ cuboids on the nickel foam were heated for 2 h in Ar and H_2/Ar atmosphere, respectively.

Supplementary Table 1 | The HER activities of the as-achieved $MoNi_4$ electrocatalyst and reported electrocatalysts.

Electrocatalysts	Onset overpotential (mV)	Overpotential (mV) at 10 mA/cm^2	Tafel slope (mV/decade)	TOF (s^{-1})	Electrolyte
**$MoNi_4$ (this work)**	**0**	**15**	**30**	**0.4 (50 mV)**	**1 M KOH**
NiO/Ni heterostructures[1]	0	30	51	—	1 M KOH
Co-Mo-S_x chalcogels[2]	60	210 mV at 5 mA/cm^2	—	—	0.1 M KOH
	100	235 mV at 5 mA/cm^2	—	—	0.1 M $HClO_4$
MoP[3]	50	130	48	—	1 M KOH
Co@N-doped CNTs[4]	140	370	>69	—	1 M KOH
Pt nanowires/single layer $Ni(OH)_2$ nanosheets[5]	5	98 mV at 5 mA/cm^2	—	—	1 M KOH
MoC_x nano-octahedrons[6]	80	151	59	—	1 M KOH
	25	142	53	—	0.5 M H_2SO_4
Pt_3Ni_3 nanowires[7]	0	50	—	—	1 M KOH
Cr_2O_3/NiO/Ni hybrid[8]	0	30	—	—	1 M KOH
Co/Co_3O_4 core/shell nanosheets[9]	30	95	44	—	1 M KOH
c-$CoSe_2$[10]	80	200	85	—	1 M KOH
Mo_xC/Ni@N-doped carbon[11]	20	126	—	—	1 M KOH
MoB[12]	150	220	59	—	1 M KOH
Ni-Mo nanopowders[13]	20	90	—	—	1 M KOH
N,P-doped Mo_2C@carbon nanospheres[14]	0	50	71	3.71×10^{-3} (10 mV)	1 M KOH
WC nanocrystals/CNTs[15]	45	150	106	—	0.1 M KOH
$Zn_{0.3}Co_{2.7}S_4$ polyhedra[16]	40	100	48	—	0.1 M KOH
CoP nanowires/carbon cloth[17]	150	240	129	—	1 M KOH
Ni/CeO_2/CNTs[18]	30	91	—	—	1 M KOH
$Co(OH)_2$/PANI hybrid[19]	50	90	92	—	1 M KOH
Pyrite-type CoPS nanowires[20]	15	48	48	—	0.5 M H_2SO_4
Strained MoS_2 nanosheets[21]	50	170	60	0.16	0.5 M H_2SO_4
W_2C nanoparticles[22]	50	123	45	—	0.5 M H_2SO_4
MoSSe/$NiSe_2$ foam[23]	20	69	42	—	0.5 M H_2SO_4
Pyrite-type CoPS/CNTs[24]	0	48	55	—	0.5 M H_2SO_4
Ni-doped carbon[25]	5	34	41	—	0.5 M H_2SO_4
Co-doped graphene[26]	30	180	82	0.22 (50 mV)	0.5 M H_2SO_4
Mo_2C/carbon/graphene[27]	0	34	34	—	0.5 M H_2SO_4
$WO_{2.9}$ powder[28]	10	70	50	8.0 (100 mV)	0.5 M H_2SO_4
WO_2/carbon nanowires[29]	10	58	46	—	0.5 M H_2SO_4

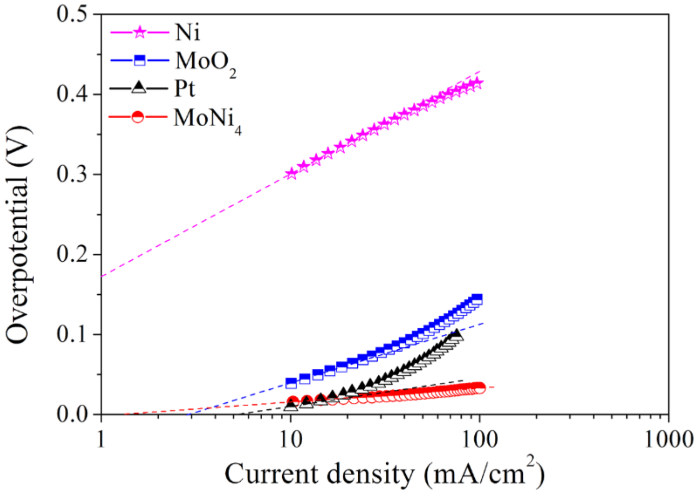

Supplementary Figure 30 | The exchange current densities of the electrocatalysts.

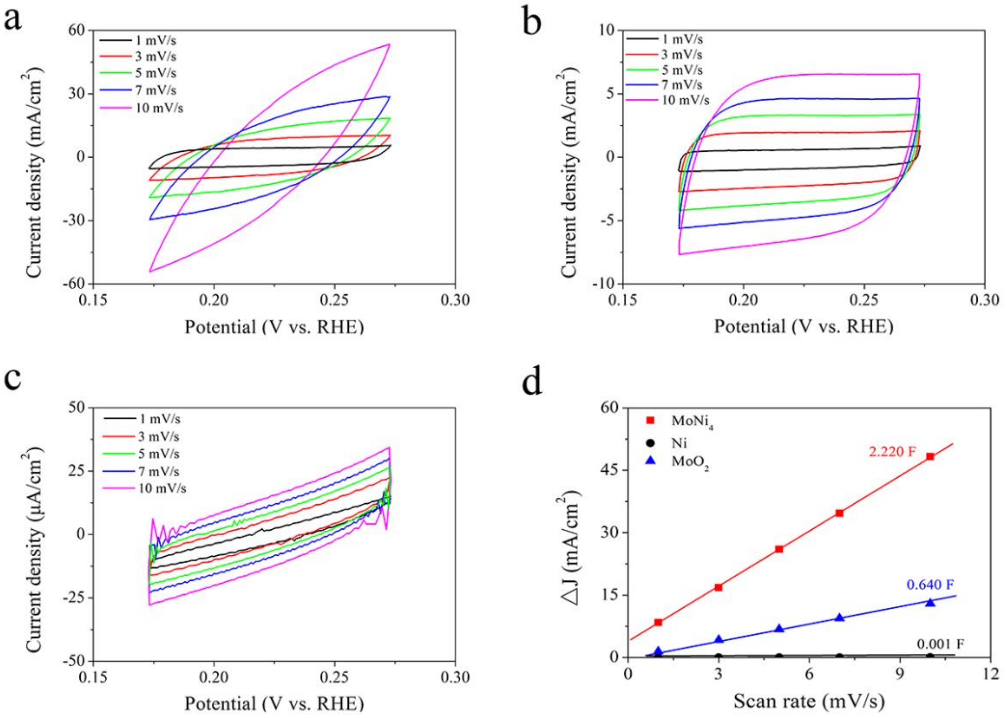

Supplementary Figure 31 | The Cps of the Ni nanosheets, the MoO_2 cuboids, and $MoNi_4/MoO_2$@Ni were assessed utilizing a series of cyclic voltammetry (CV) cycles at different scan rates. Cyclic voltammograms at different scan rates in the region between 0.175 and 0.275 V (vs. RHE) were recorded: a) $MoNi_4/MoO_2$@Ni, b) the MoO_2 cuboids, and c) the Ni nanosheets. d) The differences in current densities (ΔJ = Ja - Jc) at 0.225 V (vs. RHE) plotted against the scan rates fit to a linear regression; Cp is 1/2 of the slope.

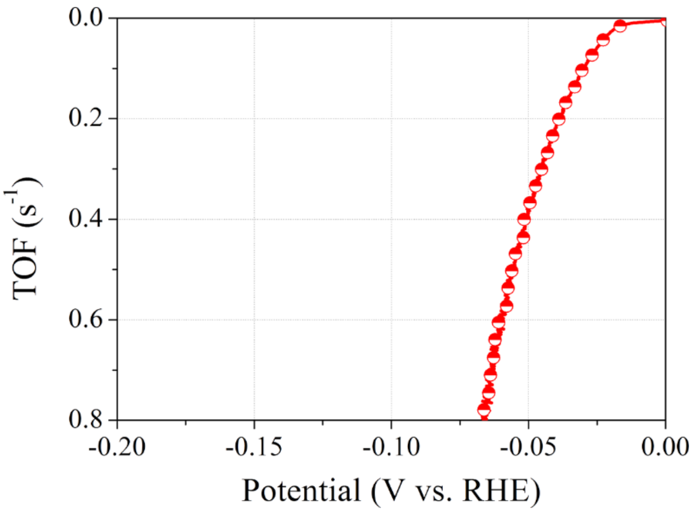

Supplementary Figure 32 | The calculated turnover frequency (TOF) for $MoNi_4/MoO_2$@Ni on the nickel foam.

Calculation of TOF: The TOF (in s^{-1}) were calculated with the following equation:

TOF = I/(2NF)

I: current (in A) during the linear sweep measurement.

The factor ½ arrives by taking into account that two electrons are required to form one hydrogen molecule from two protons.

N: number of active sites (in mol): cyclic voltammetry measurements were conducted from -0.87 V to -0.77 V at 1 mV/s in 1 M KOH solution. The absolute components of the voltammetric charges (cathodic and anodic) tested during one CV cycle were calculated. Assuming a one electron redox process, this absolute charge was divided by two. The obtained value was then divided by the Faraday constant to get the number of active sites of the catalysts.

F: Faraday constant (in C/mol).

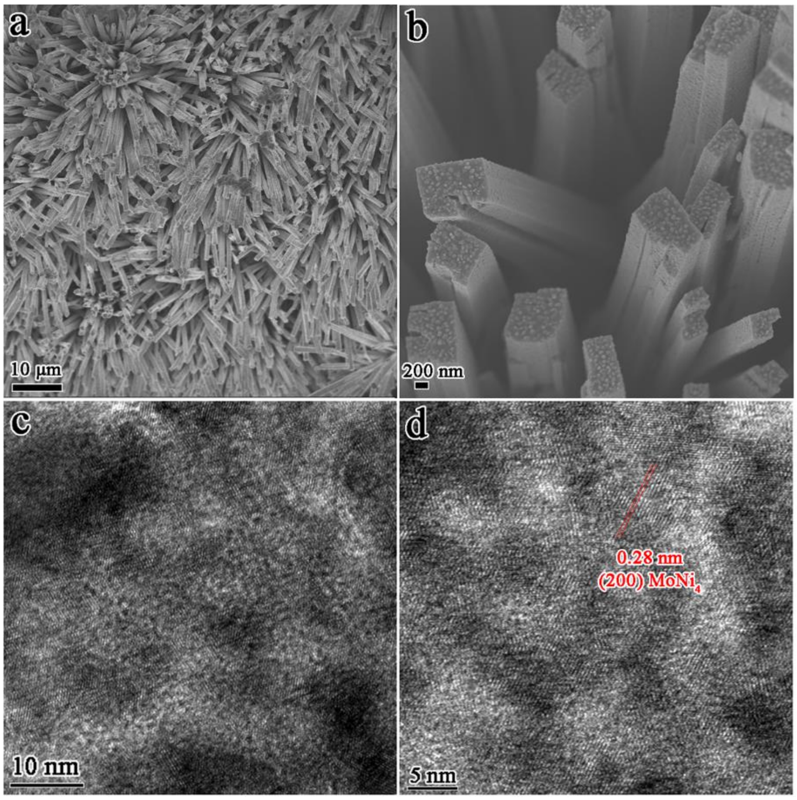

Supplementary Figure 33 | Morphology. (a and b) SEM and (c and d) TEM images of $MoNi_4/MoO_2$@Ni after a long-term HER stability test in a 1 M KOH electrolyte.

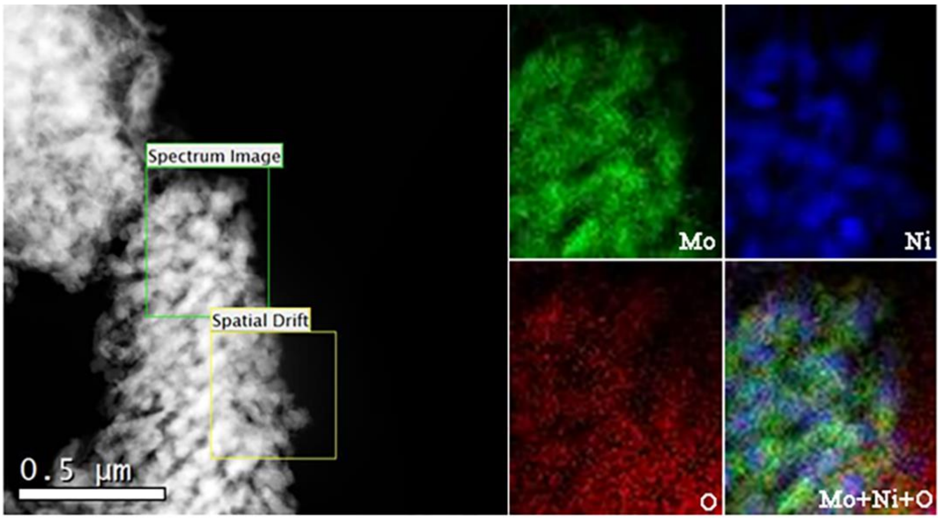

Element	Line	KRatio	Correction	Overvoltage	Weight	Atoms %
O	Ka	0.008 ± 0.0001	1.0000	0.00	0.01 ± 0.00	3.38 ± 0.06
Ni	Ka	0.657 ± 0.0018	1.0000	0.00	0.66 ± 0.00	73.67 ± 0.21
Mo	Ka	0.335 ± 0.0041	1.0000	0.00	0.33 ± 0.00	22.95 ± 0.28

Supplementary Figure 34 | TEM and corresponding elemental mapping images of $MoNi_4/MoO_2$@Ni after a long-term stability test.

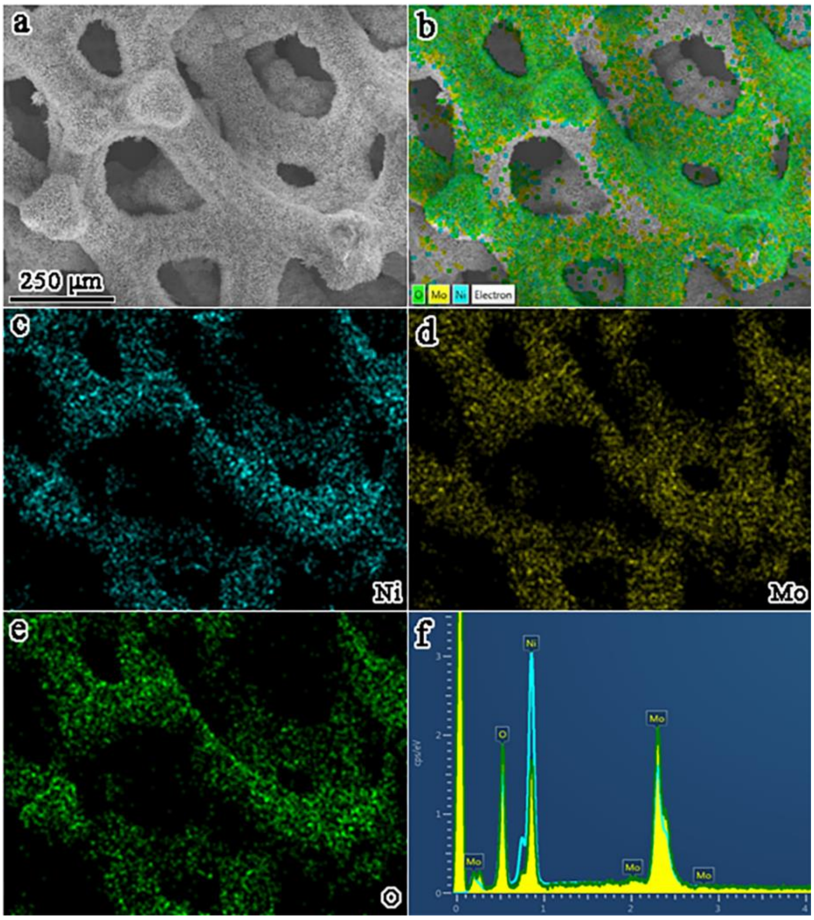

Supplementary Figure 35 | Morphology and chemical composition analyses. (a) SEM image of the $MoNi_4/MoO_2$@Ni; corresponding elemental mapping images: (b) Ni+Mo+O, (c) Ni, (d) Mo and (e) O; (f) EDX analysis of $MoNi_4/MoO_2$@Ni after long-term HER stability tests in a 1 M KOH electrolyte.

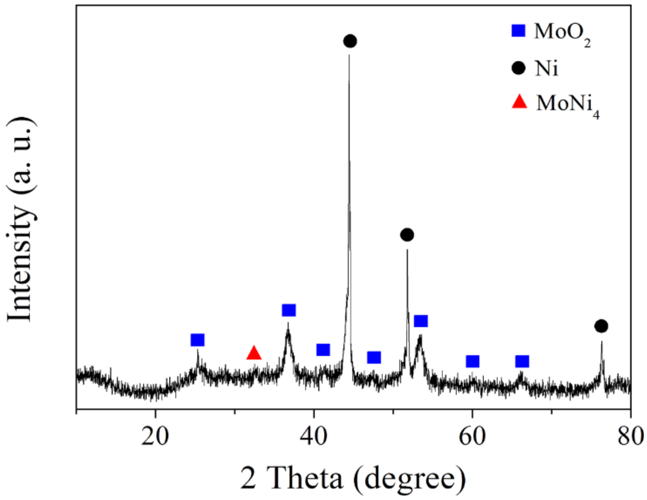

Supplementary Figure 36 | The XRD pattern of $MoNi_4/MoO_2$@Ni after long-term stability tests.

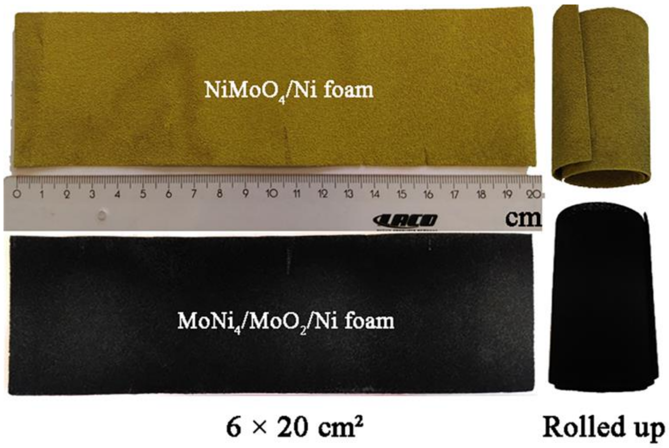

Supplementary Figure 37 | Photographs of the $NiMoO_4$ cuboids and $MoNi_4/MoO_2$@Ni on the nickel foam.

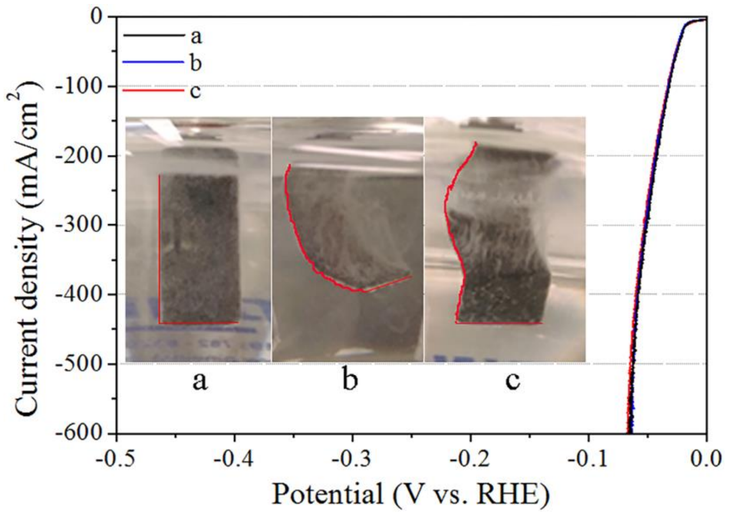

Supplementary Figure 38 | The polarization curves of $MoNi_4/MoO_2$@Ni with different deformations, as shown in the inset.

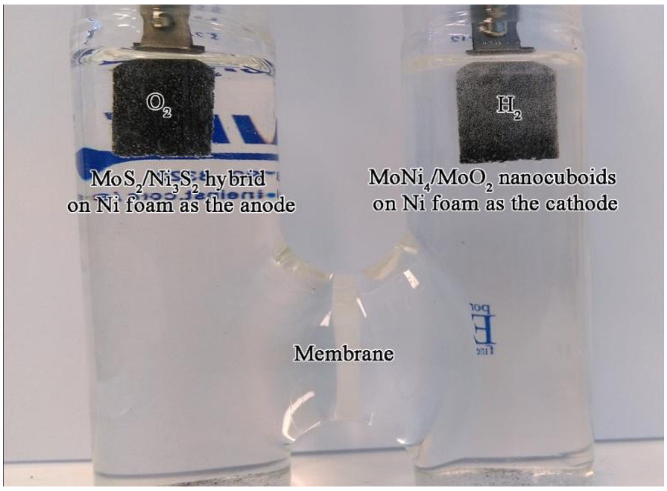

Supplementary Figure 39 | A functional alkaline electrolyzer in a 1 M KOH aqueous solution using $MoNi_4/MoO_2$@Ni as the cathode and the reported MoS_2/Ni_3S_2 hybrid on the nickel foam as the anode.

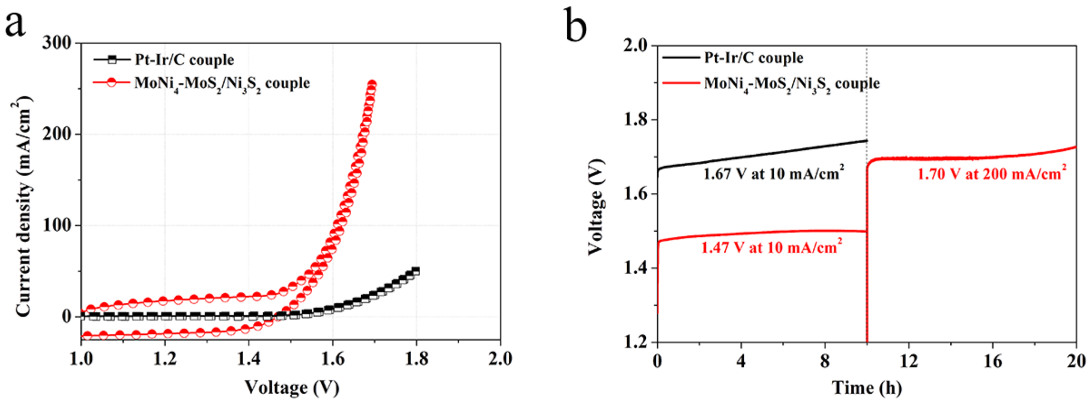

Supplementary Figure 40 | Electrochemical tests. (a) CV curves and (b) long term durability tests of the Pt-Ir/C couple and $MoNi_4$-MoS_2/Ni_3S_2 couple in a water-alkali electrolyzer. Electrolyte: 1 M KOH solution; scan rate: 1 mV s^{-1}.

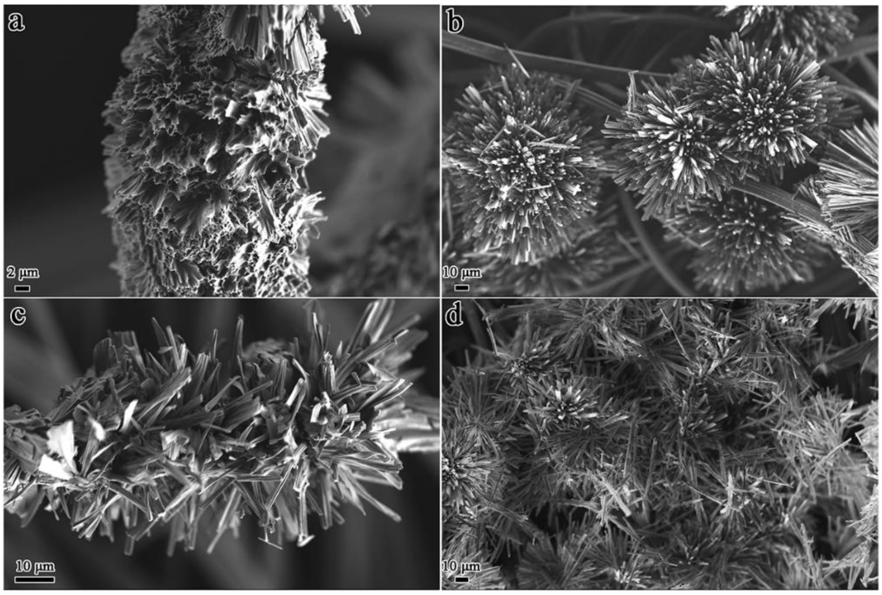

Supplementary Figure 41 | Morphology. SEM images of (a) MoO_3 nanosheets and (b) $NiMoO_4$ cuboids on the carbon cloth; SEM images of (c) MoO_2 nanosheets and (d) the $MoNi_4$ electrocatalyst supported by the MoO_2 cuboids on the carbon cloth.

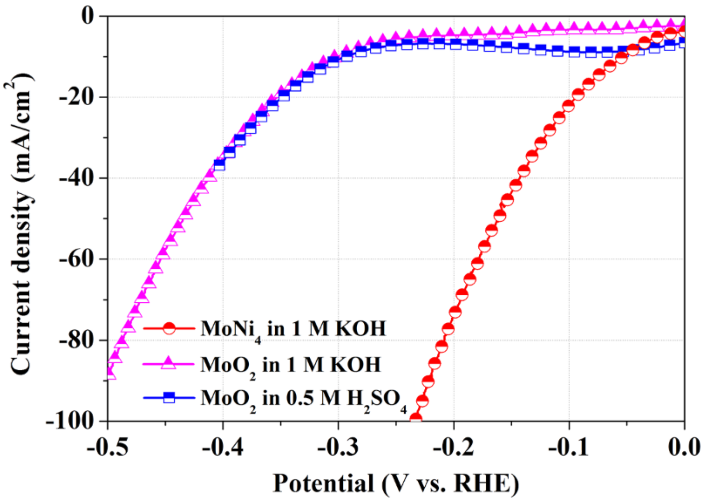

Supplementary Figure 42 | (a) Polarization curves of the MoO_2 nanosheets and the $MoNi_4$ electrocatalyst supported by the MoO_2 cuboids on the carbon cloth in different electrolytes.

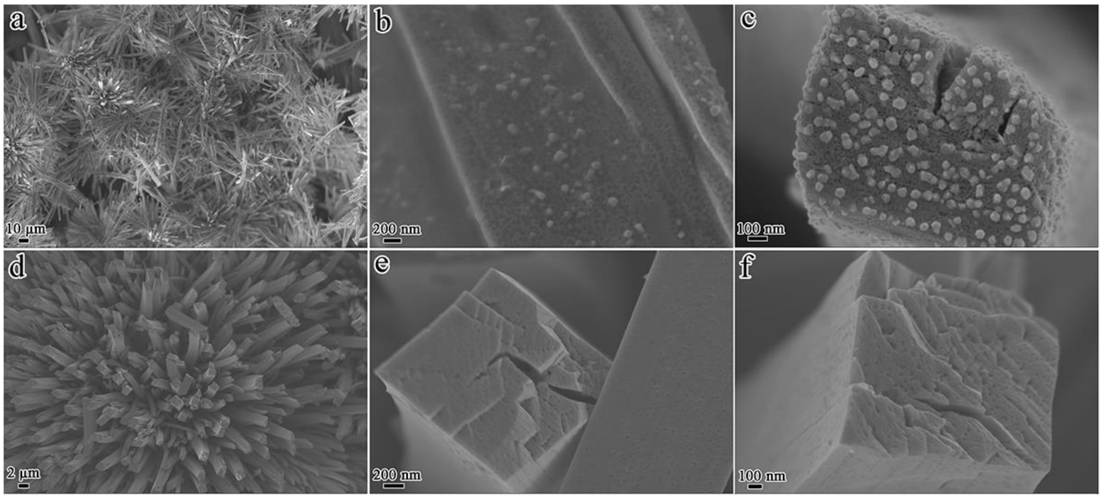

Supplementary Figure 43 | Morphology. SEM images of the $MoNi_4/MoO_2@C$: a-b) before and c-d) after etching $MoNi_4$ in 2 M H_2SO_4 solution. Accordingly, the weight content of $MoNi_4$ in $MoNi_4/MoO_2$ is estimated to be 9.5 wt%.

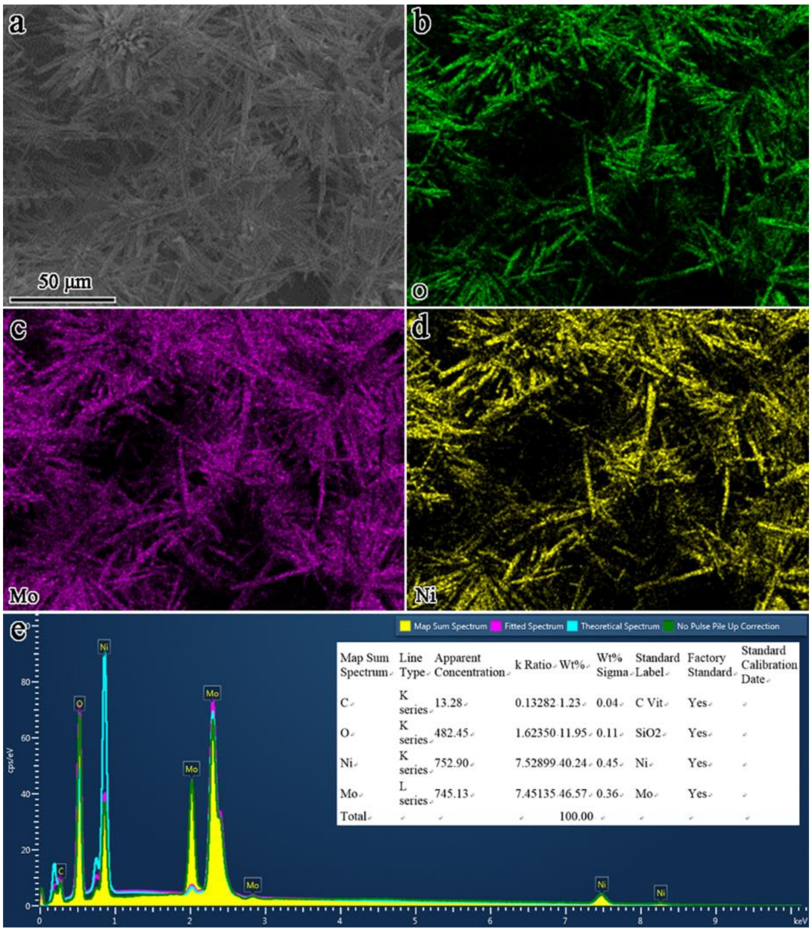

Supplementary Figure 44 | Chemical composition analyses of $MoNi_4/MoO_2@C$ before etching $MoNi_4$. a) SEM image; b-d) corresponding elemental mapping images of Ni, Mo and O; e) related EDX analysis.

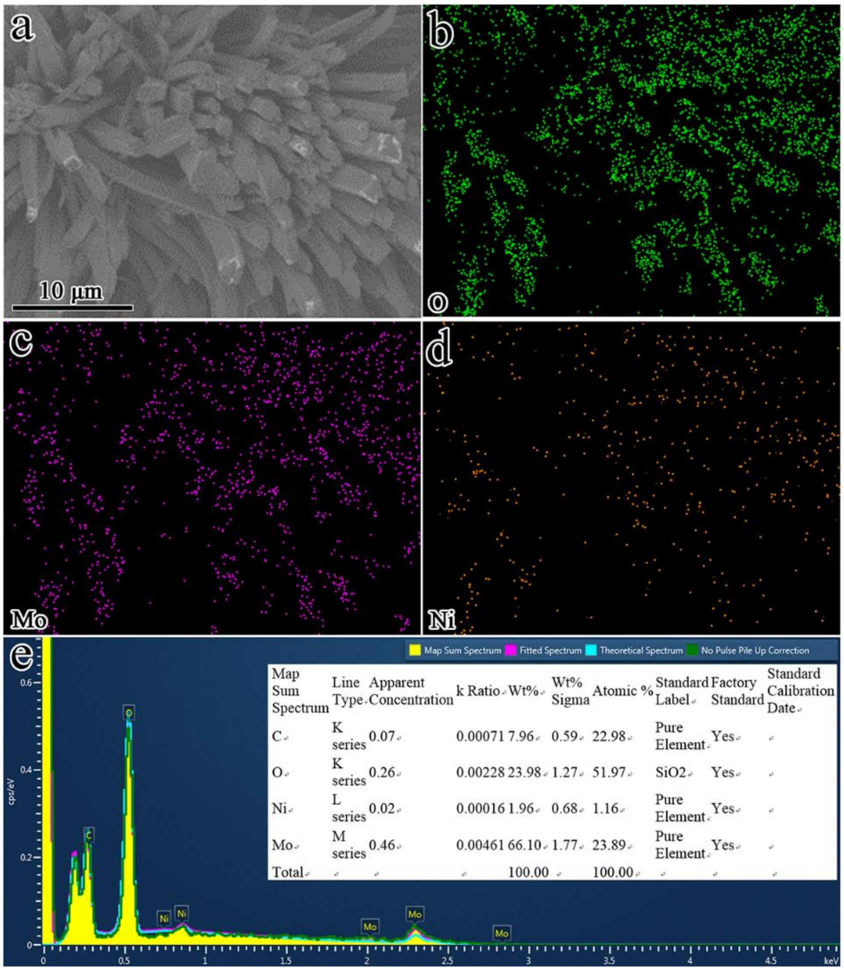

Supplementary Figure 45 | Chemical composition analyses of $MoNi_4/MoO_2$@C after etching $MoNi_4$. a) SEM image; b-d) corresponding elemental mapping images of Ni, Mo and O; e) related EDX analysis.

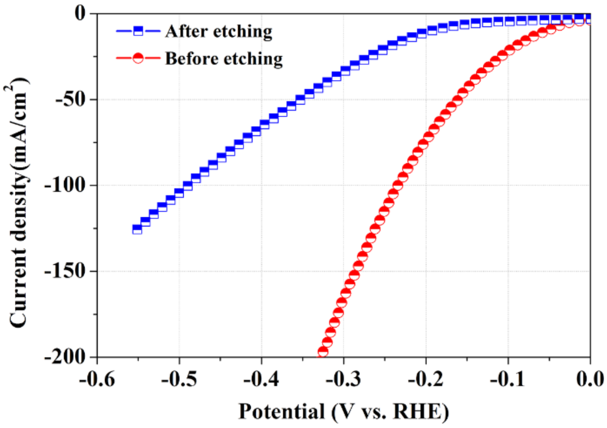

Supplementary Figure 46 | The polarization curves of the $MoNi_4/MoO_2$@C before

and after etching the $MoNi_4$ in 2 M H_2SO_4 solution.

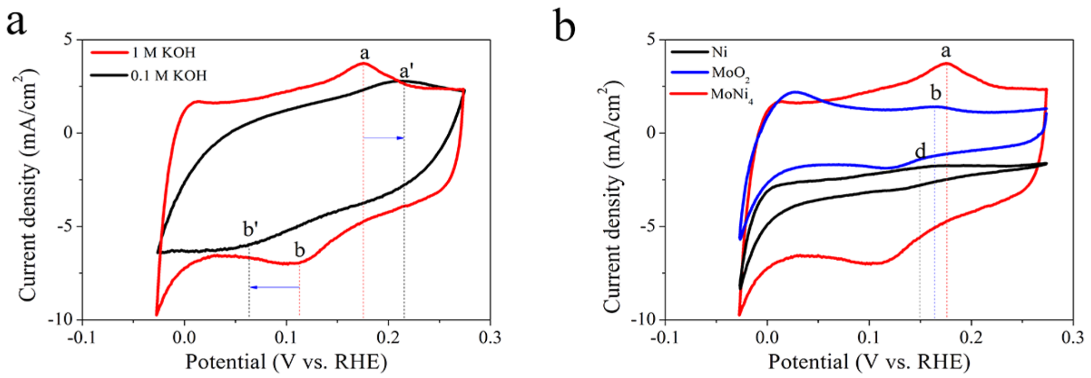

Supplementary Figure 47 | (a) CV curves of the $MoNi_4$ electrocatalyst supported by the MoO_2 cuboids on the nickel foam in different electrolytes; (b) CV curves of $MoNi_4/MoO_2$@Ni, the Ni nanosheets and the MoO_2 cuboids on the nickel foam in a 1 M KOH electrolyte. Scan rate: 1 mV s^{-1}.

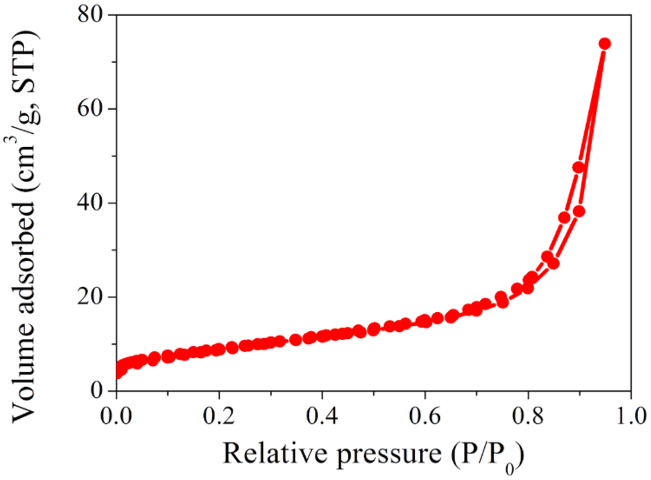

Supplementary Figure 48 | Nitrogen adsorption/desorption isotherm of the $MoNi_4$ electrocatalyst supported by the MoO_2 cuboids. The specific surface area of the $MoNi_4$ electrocatalyst supported by the MoO_2 cuboids was approximately 32 m^2/g. The specific surface area of the commercial Pt catalyst is 120 m^2 g^{-1}.

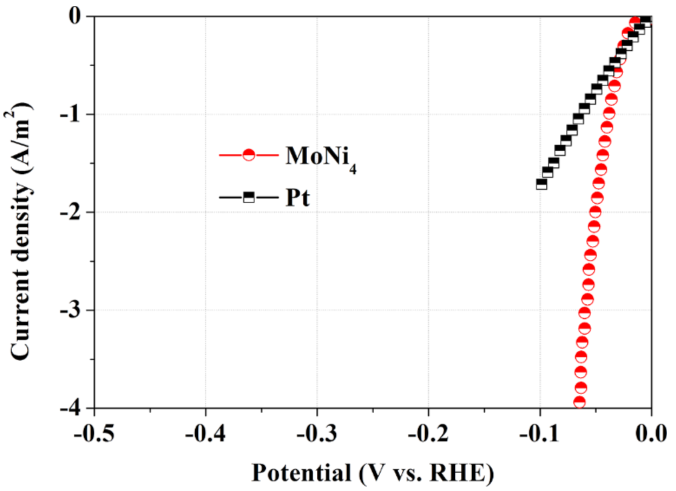

Supplementary Figure 49 | The normalized polarization curves of $MoNi_4/MoO_2$@Ni and the Pt catalyst on the nickel foam. The weight content of $MoNi_4$ in $MoNi_4/MoO_2$ was estimated to be 9.5% and the loading weight of $MoNi_4/MoO_2$ on nickel foam was approximately 43.4 mg. Thus, the specific surface area of $MoNi_4$ was about 0.1319 m^2. The HER current density of $MoNi_4$ is then normalized by its specific surface area of 0.1319 m^2.

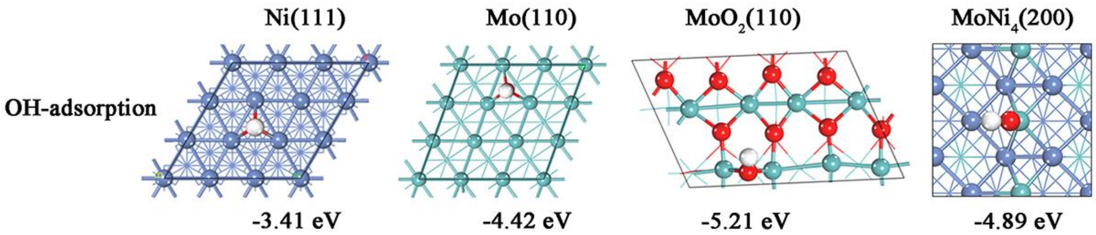

Supplementary Figure 50 | The as-built catalyst models for the DFT calculations. Blue balls: Ni; Aqua balls: Mo; Red balls: O.

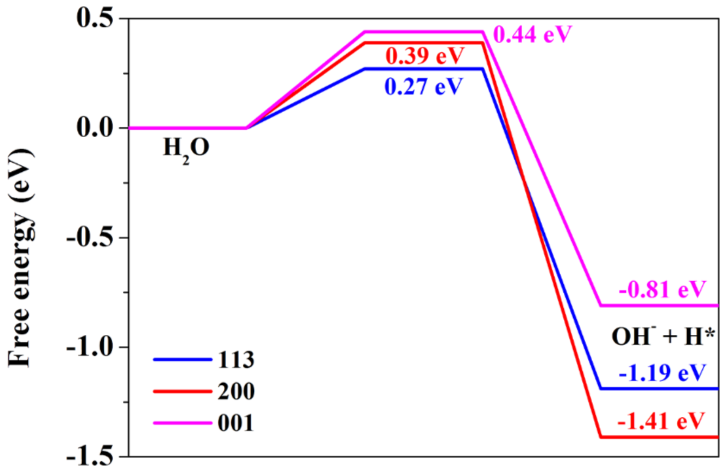

Supplementary Figure 51 | The calculated adsorption free energy diagram for the Volmer step on different facets of $MoNi_4$.

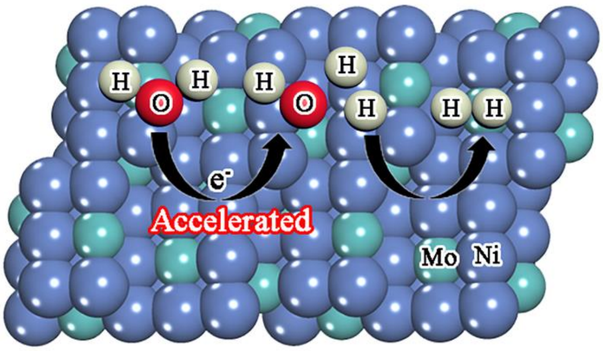

Supplementary Figure 52 | The proposed HER mechanism on the $MoNi_4$ electrocatalyst.

Supplementary References

1. Gong M, *et al.* Nanoscale nickel oxide/nickel heterostructures for active hydrogen evolution electrocatalysis. *Nat. Commun.* **5**, 4695-4700 (2014).
2. Staszak-Jirkovsky J, et al. Design of active and stable Co-Mo-S_x chalcogels as pH-universal catalysts for the hydrogen evolution reaction. *Nat. Mater.* **15**, 197-203 (2016).
3. Xiao P, *et al.* Molybdenum phosphide as an efficient electrocatalyst for the hydrogen evolution reaction. *Energy Environ. Sci.* **7**, 2624-2629 (2014).
4. Zou X, *et al.* Cobalt-embedded nitrogen-rich carbon nanotubes efficiently catalyze hydrogen evolution reaction at all pH values. *Angew. Chem.-Int. Edit.* **126**, 4461-4465

(2014).

5. Yin H, *et al.* Ultrathin platinum nanowires grown on single-layered nickel hydroxide with high hydrogen evolution activity. *Nat. Commun.* **6**, 6430-6437 (2015).
6. Wu HB, Xia BY, Yu L, Yu X-Y, Lou XW. Porous molybdenum carbide nano-octahedrons synthesized via confined carburization in metal-organic frameworks for efficient hydrogen production. *Nat. Commun.* **6**, 6512-6519 (2015).
7. Wang P, Jiang K, Wang G, Yao J, Huang X. Phase and interface engineering of platinum–nickel nanowires for efficient electrochemical hydrogen evolution. *Angew. Chem.-Int. Edit.* **128**, 13051-13055 (2016).
8. Gong M, *et al.* Blending Cr_2O_3 into a NiO-Ni electrocatalyst for sustained water splitting. *Angew. Chem.-Int. Edit.* **54**, 1-6 (2015).
9. Yan X, Tian L, He M, Chen X. Three-dimensional crystalline/amorphous Co/Co_3O_4 core/shell nanosheets as efficient electrocatalysts for the hydrogen evolution reaction. *Nano Lett.* **15**, 6015-6021 (2015).
10. Chen P, *et al.* Phase-transformation engineering in cobalt diselenide realizing enhanced catalytic activity for hydrogen evolution in an alkaline medium. *Adv. Mater.* **28**, 7527-7532 (2016).
11. Wang S, *et al.* Molybdenum carbide-modified nitrogen-doped carbon vesicle encapsulating nickel nanoparticles: a highly efficient, low-cost catalyst for hydrogen evolution reaction. *J. Am. Chem. Soc.* **137**, 15753–15759 (2015).
12. Vrubel H, Hu X. Molybdenum boride and carbide catalyze hydrogen evolution in both acidic and basic solutions. *Angew. Chem.-Int. Edit.* **124**, 12875-12878 (2012).
13. McKone JR, Sadtler BF, Werlang CA, Lewis NS, Gray HB. Ni-Mo nanopowders for efficient electrochemical hydrogen evolution. *ACS Catal.* **3**, 166-169 (2013).
14. Chen Y-Y, *et al.* Pomegranate-like N,P-doped Mo_2C@C nanospheres as highly active electrocatalysts for alkaline hydrogen evolution. *ACS Nano* **10**, 8851–8860 (2016).
15. Fan X, Zhou H, Guo X. WC nanocrystals grown on vertically aligned carbon nanotubes: an efficient and stable electrocatalyst for hydrogen evolution reaction. *ACS Nano* **9**, 5125-5134 (2015).
16. Huang Z-F, *et al.* Hollow cobalt-based bimetallic sulfide polyhedra for efficient all-pH-

value electrochemical and photocatalytic hydrogen evolution. *J. Am. Chem. Soc.* **138**, 1359-1365 (2016).

17. Tian J, Liu Q, Asiri AM, Sun X. Self-supported nanoporous cobalt phosphide nanowire arrays: an efficient 3D hydrogen-evolving cathode over the wide range of pH 0-14. J. *Am. Chem. Soc.* **136**, 7587-7590 (2014).
18. Weng Z, *et al.* Metal/oxide interface nanostructures generated by surface segregation for electrocatalysis. *Nano Lett.* **15**, 7704-7710 (2015).
19. Feng J-X, *et al.* $Co(OH)_2$@PANI hybrid nanosheets with 3D networks as nigh-performance electrocatalysts for hydrogen evolution reaction. *Adv. Mater.* **27**, 7051-7057 (2015).
20. Caban-Acevedo M, *et al.* Efficient hydrogen evolution catalysis using ternary pyrite-type cobalt phosphosulphide. *Nat. Mater.* **14**, 1245-1251 (2015).
21. Li H, *et al.* Activating and optimizing MoS_2 basal planes for hydrogen evolution through the formation of strained sulphur vacancies. *Nat. Mater.* **15**, 48-53 (2016).
22. Gong Q, *et al.* Ultrasmall and phase-pure W_2C nanoparticles for efficient electrocatalytic and photoelectrochemical hydrogen evolution. *Nat. Commun.* **7**, 13216-13223 (2016).
23. Zhou H, *et al.* Efficient hydrogen evolution by ternary molybdenum sulfoselenide particles on self-standing porous nickel diselenide foam. *Nat. Commun.* **7**, 12765-12771 (2016).
24. Liu W, *et al.* A highly active and stable hydrogen evolution catalyst based on pyrite-structured cobalt phosphosulfide. *Nat. Commun.* **7**, 10771-10779 (2016).
25. Fan L, *et al.* Atomically isolated nickel species anchored on graphitized carbon for efficient hydrogen evolution electrocatalysis. *Nat. Commun.* **7**, 10667-10673 (2016).
26. Fei H, *et al.* Atomic cobalt on nitrogen-doped graphene for hydrogen generation. *Nat. Commun.* **6**, 8668-8675 (2015).
27. Li J-S, *et al.* Coupled molybdenum carbide and reduced graphene oxide electrocatalysts for efficient hydrogen evolution. *Nat. Commun.* **7**, 11204-11211 (2016).
28. Li YH, *et al.* Local atomic structure modulations activate metal oxide as electrocatalyst for hydrogen evolution in acidic water. *Nat. Commun.* **6**, 8064-8070 (2015).
29. Wu R, Zhang J, Shi Y, Liu D, Zhang B. Metallic WO_2-carbon mesoporous nanowires as highly efficient electrocatalysts for hydrogen evolution reaction. *J. Am. Chem. Soc.* **137**,

6983–6986, (2015).
